# Low-intensity pulsed ultrasound stimulated hydrogel-polylactic acid composite scaffolds: a dual-cue approach for enhanced rotator cuff healing

**DOI:** 10.1093/rb/rbag112

**Published:** 2026-06-09

**Authors:** Lan Jiang, Yangying Duan, Yong Zhang, Dong Wang, Haitao Ran

**Affiliations:** Department of Ultrasound, The Second Affiliated Hospital of Chongqing Medical University, Chongqing 400010, China; Chongqing Key Laboratory of Ultrasound Molecular Imaging and Therapy, The Second Affiliated Hospital of Chongqing Medical University, Chongqing 400000, China; Department of Ultrasound, The First Affiliated Hospital of Chongqing Medical University, Chongqing 400016, China; Department of Ultrasound, Sichuan Provincial People’s Hospital, School of Medicine, University of Electronic Science and Technology of China, Chengdu 610072, China; Department of Ultrasound, The First Affiliated Hospital of Chongqing Medical University, Chongqing 400016, China; Department of Ultrasound, Sichuan Provincial People’s Hospital, School of Medicine, University of Electronic Science and Technology of China, Chengdu 610072, China; Department of Ultrasound, The Second Affiliated Hospital of Chongqing Medical University, Chongqing 400010, China; Chongqing Key Laboratory of Ultrasound Molecular Imaging and Therapy, The Second Affiliated Hospital of Chongqing Medical University, Chongqing 400000, China

**Keywords:** low-intensity pulsed ultrasound, acoustic stimulation, composite scaffold, tendon–bone interface regeneration

## Abstract

Rotator cuff tear is a prevalent musculoskeletal condition. The complex soft-to-hard transition at the tendon–bone interface (TBI) makes the healing process more challenging than in homogeneous tissues. In this study, low-intensity pulsed ultrasound (LIPUS), a non-invasive modality for bone repair, was employed as an external stimulus in combination with tissue engineering. A composite scaffold, designated as BMP-2/bFGF@GM-PLA, was developed by incorporating a gelatin-methacryloyl (GM) hydrogel loaded with bone morphogenetic protein-2 (BMP-2) and basic fibroblast growth factor (bFGF) into polylactic acid (PLA) electrospun fibers. Under LIPUS stimulation, the BMP-2/bFGF@GM-PLA scaffold effectively promotes migration, osteogenic and tenogenic differentiation of the bone marrow mesenchymal stem cells through synergistic mechanical and chemical cues. *In vivo*, the combined system of BMP-2/bFGF@GM-PLA implantation and post-operative LIPUS treatments significantly enhances healing in rat models of rotator cuff tear, as evidenced by enhanced biomechanical properties, accelerated bone defect repair and improved histological structure of the TBI. This study proposes a novel approach that combines LIPUS stimulus with acoustic-responsive biomaterial scaffolds, thereby coordinating mechanical and chemical cues to facilitate rotator cuff healing.

## Graphical abstract

**Graphical Abstract:**
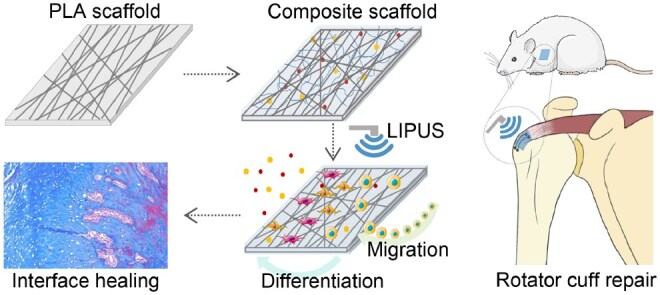

## Introduction

Rotator cuff tear is a leading cause of shoulder pain and dysfunction, affecting over 20% of the general population, and typically occurs at the tendon–bone interface (TBI) [1]. When conservative treatment is ineffective, surgical repair is a viable therapeutic option for alleviating pain and restoring shoulder function [2]. One challenge in rotator cuff repair is the poor healing of the TBI, with post-operative retear rates reported to range from 26% for small and medium tears (<3 cm) to 94% for large and massive tears (>3 cm) [3]. The native TBI exhibits a complex histological structure comprising tendon, fibrocartilage, mineralized fibrocartilage and bone as transitional regions [4], making its healing more challenging than that of a single tissue type. To reduce the post-operative retears, enhancing the healing quality of the TBI and reconstructing the interface with biomimetic structure are of critical importance.

Interface tissue engineering has emerged as a key strategy to facilitate the regeneration of soft-to-hard tissue interfaces and improve postoperative healing outcomes in rotator cuff tears. The three major approaches in tissue engineering include stem cells, growth factors and biomaterial scaffolds [5]. Intramedullary tunnels are typically drilled at the greater tuberosity of the humerus during rotator cuff repair surgery to facilitate tendon reattachment. This procedure also facilitates the influx of bone marrow mesenchymal stem cells (BMSCs) into the repair site, providing a reliable endogenous source of stem cells for the healing process [6]. Growth factors, which act as chemical cues, have been proven to promote the growth and differentiation of mesenchymal stem cells (MSCs), thereby improving the healing outcomes of rotator cuff tears [7].

Among these approaches, the design and fabrication of biomimetic scaffolds are particularly crucial. An ideal rotator cuff repair scaffold needs to reconcile biocompatibility, mechanical properties and drug delivery. Macroscopically, the scaffold should bridge the tear gap and share the load at the repair site; microscopically, it should mimic the TBI microenvironment to promote interfacial tissue healing [3]. 3D printing techniques are commonly used to fabricate customized scaffolds, yet 3D-printed scaffolds typically exhibit limited flexibility due to their thickness, which prevents them from fully conforming to the curved surface of the rotator cuff [8]. Decellularized and hydrogel scaffolds exhibit favorable conformability and provide a microenvironment that closely mimics the extracellular matrix (ECM). Nevertheless, these scaffolds generally lack the mechanical strength required to withstand the stresses at the TBI [9, 10]. The use of allografts or heterografts also poses risks of immune rejection and infection. Electrospun fibrous scaffolds, which possess similarities to the microstructure and mechanical properties of natural tendon tissue, are particularly suitable for tendon tissue engineering [3]. Synthetic biodegradable polymers, such as polylactic acid (PLA), are frequently used in electrospinning. A limitation of electrospun scaffolds is the high electric field required during fabrication, which can compromise the bioactivity of loaded molecules [11]. Overall, accurate reproduction of the native TBI structure and simultaneous promotion of osseous and tendinous healing remain major challenges in rotator cuff scaffold design [12]. The biological complexity of rotator-cuff regeneration necessitates more refined strategies to deliver superior TBI healing.

External stimuli, including acoustic, optical, electrical, magnetic and mechanical stimuli, can remotely control various biological processes [13]. Low-intensity pulsed ultrasound (LIPUS) is a non-invasive, FDA approved modality for promoting healing in acute fractures and delayed or non-union fractures. Studies indicate that LIPUS promotes osteogenic differentiation of MSCs via multiple signaling pathways [14, 15]. In tendon/ligament injuries, LIPUS is reported to increase collagen deposition, matrix alignment and M2 polarization, accelerating tissue healing [16]. In interface repair, LIPUS is shown to enhance adipose-derived stromal cell survival and differentiation to improve TBI healing [17]. The technique delivers low-energy and high-frequency sound waves to tissues, inducing therapeutic effects through non-thermal mechanical effect, cavitation, microstreaming and acoustic radiation force. In theory, this stimulus can induce mechanical vibration in acoustic-active materials with moderate Young’s modulus and low damping characteristics like PLA [18], enabling these materials to vibrate under the mechanical effect of ultrasound and thereby facilitate the propagation and concentration of ultrasound energy within the scaffolds. Studies have verified that scaffolds composed of PLA exhibit mechanical vibrations with large amplitudes and pulse widths when subjected to ultrasound, thereby further promoting cell migration and osteogenic differentiation [19, 20]. These findings indicate that LIPUS can remotely control acoustic-responsive scaffolds, offering a new perspective on the combined system as a promising strategy for TBI healing in rotator cuff tears.

In the present study, a biomimetic composite scaffold designated as BMP‑2/bFGF@GM‑PLA was fabricated by incorporating growth factor‑loaded gelatin‑methacryloyl (GM) hydrogel into a PLA electrospun mat. The PLA electrospun fibers, which served as the structural framework of the rotator cuff scaffold, were capable of responding to remote LIPUS stimulation via mechanical vibration. The dual‑cue strategy combining LIPUS with BMP‑2/bFGF@GM‑PLA was hypothesized to achieve synergistic effects on cell proliferation and osteogenic differentiation, outperforming single‑factor approaches. By innovatively using LIPUS as an external stimulus to integrate mechanical activation with the biochemical cues of the scaffold, this study provides a novel strategy for enhancing TBI regeneration.

## Materials and methods

### Fabrication of PLA scaffolds

Referred to previous researches [21], a 12% (w/v) electrospinning solution was prepared by dissolving 1.2 g of PLA (Mw: 260–360 kDa, Daigang biomaterial, China) in 10 mL of 1,1,1,3,3,3-hexafluoro-2-propanol (HFIP, 99.5%, Macklin, China) and stirring magnetically for 12 h until the polymer crystal dissolved. The PLA solution was loaded into a 10 mL syringe with the flow rate set at 3 mL/h, and a high voltage of +15 kV was applied to the needle (22G). The rotated collector was wrapped around with aluminum foil and placed at 10 cm from the needle tip to receive the fibers. The electrospun mats were detached and vacuum dried for 8 h, cut into 5 mm × 5 mm squares, sterilized in 75% ethanol (Macklin, China) for 1 h and washed with phosphate buffer saline (PBS, Boster, China) before being utilized as PLA scaffolds.

### Fabrication of BMP-2/bFGF@GM-PLA scaffolds

About 25 mg of lithium phenyl-2,4,6-trimethylbenzoylphosphinate (LAP) was dissolved in 10 mL PBS to prepare a 0.25% (w/v) photoinitiator solution. GM (PR-002, EFL-Tech, China) was added to the solution, stirred at 37°C in the dark for 1 h and sterilized through a 0.22 μm syringe filter to obtain final concentrations of 4%, 6% and 8% (w/v). Recombinant bone morphogenetic protein-2 (BMP-2) and basic fibroblast growth factor (bFGF, Beyotime, China) were blended with the precursor solution to achieve a concentration of 2 μg/mL. The composite scaffold of BMP-2/bFGF@GM-PLA was formed based on previous research [22]. Specifically, the PLA scaffolds were fully soaked in 10 μL of the BMP-2 and bFGF loaded precursor solution overnight at 4°C, then photo-crosslinked under 405 nm UV light for 16–18 s to form 0.5 mm-thick composite scaffolds.

### Characterization of PLA and BMP-2/bFGF@GM-PLA scaffolds

The water contact angle was measured using sessile drop method by video contact angle meter (JY-82C, Chengde DingSheng, China). The morphology of the scaffolds was observed by scanning electron microscopy (SEM, Regulus 8230, Hitachi Ltd., Japan). The scaffolds were treated with or without LIPUS stimulation at 100 mW/cm^2^, 1 MHz, 20% duty cycle for 10 min. The samples were frozen at −80°C for 2 h and vacuum freeze-dried for 18 h. To observe the cross-sections, the samples underwent liquid nitrogen brittle fracture. The samples were coated with gold and placed in the vacuum chamber at an accelerating voltage of 5 kV for observation. The fiber diameter and pore area were measured using ImageJ software (National Institutes of Health, USA). The surface mechanics and adhesion properties of the composite scaffold after LIPUS stimulation were further evaluated using atomic force microscope (AFM, Dimension Icon, Bruker, Germany). The probe (RTESPA-150) with a spring constant of 6 N/m was used. The mechanical properties of the scaffolds were assessed using mechanical testing machine (CMT6103, Meters Industrial Systems Co., Ltd, China). The stress–strain curve was obtained by uniaxially stretching the scaffold at speed of 1 mm/min, from which the Young’s modulus, tensile strength, fracture strain and maximum stress of the scaffolds were derived. Vibration response of the PLA scaffolds to LIPUS stimulation was evaluated using laser Doppler vibrometry (LDV, OFV-5000, Polytec, Germany). The laser beam was focused onto a reflective marker on the scaffold surface. Fast Fourier transform (FFT) analysis of the velocity signal of the laser spot yielded the frequency spectrum. The displacement amplitude was calculated by converting the velocity signal to displacement at the dominant vibration frequency.

### Cell culture on scaffolds

Rat BMSCs (Cellverse bioscience technology, China) were passaged to the fourth generation for experimental use. The complete culture medium was used to culture cells, which included α-MEM medium (Hyclone, USA), fetal bovine serum (FBS, 10%, Gibco, USA) and penicillin/streptomycin (1%, Solarbio, China). The cells were seeded onto each of the scaffolds at concentration of 1 × 10^5^ per well and allowed to attach for 15 min, before 450 μL of complete culture medium was added. The BMSCs were cultured on scaffolds in a 37°C incubator with 5% CO_2_, with the culture medium being changed every 3 days.

### LIPUS stimulation *in vitro* and cell viability assay

For the ultrasound-treated groups, LIPUS (Chattanooga, DJO, USA) was applied to the cell on scaffolds by irradiating to the bottom of the cell culture plates through coupling gel [23, 24]. The cells were then irradiated by LIPUS at different acoustic intensities at 50, 100 or 200 mW/cm^2^, and different frequencies of 1 or 3 MHz, and a duty cycle of 20% for 10 min daily. Cell counting kit-8 (CCK8, Beyotime, China) was used to determine the viability of BMSCs on PLA scaffolds exposed to LIPUS stimulation. The samples were gently washed with PBS, after which 20 μL of CCK-8 solution diluted in 180 μL of culture medium was added into each well and incubated for 2 h. The solution of each well was transferred to a 96-well plate and OD values were measured at 450 nm using a microplate reader (Thermo Fisher, USA).


Relative cell viability (%)=(ODe−ODb)/(ODc−ODb)×100%,

where ODe is the absorbance of the experimental groups, ODb is the absorbance of blank wells, ODc is the absorbance of the BMSCs cultured on PLA scaffolds without LIPUS stimulation at D1 timepoint as control.

### LIPUS-stimulated growth factors release

Growth factors release and hydrogel degradation of BMP-2/bFGF@GM-PLA scaffold under LIPUS stimulation were assessed following reported methods [19]. For the release kinetics of growth factors, BMP-2/bFGF@GM-PLA scaffolds composed of GM at concentrations of 4%, 6% and 8% (w/v) were incubated in a 24-well plate with 2 mL PBS filled at 37°C. About 1 mL of the suspension was collected and refilled with equal volume of PBS on alternate days. Both BMP-2 and bFGF in the supernatant was quantitatively analysed using Enzyme-linked immunosorbent assay kit (ELISA, Jonlnbio, China). The composite scaffolds were soaked in PBS in a 24-well plate for 12 h to reach swelling equilibrium, weighed and recorded as W0 after being drained under gravity for 30 min. The composite scaffolds were incubated and weighed on days 1, 3, 5 and 7, each recorded as W1.


Weight loss rate (%)=(W0−W1)/W0×100%.

### Cell adhesion and proliferation

The BMSCs adhesion and morphology on the scaffolds were observed by SEM after 7 days of culture with or without LIPUS stimulation. After the medium was removed, the samples were fixed with 4% glutaraldehyde (Aladdin, China) at 4°C for 2 h before gradient dehydration was performed, vacuum-dried, coated with gold observed using SEM. The biocompatibility and pro-proliferative effect of scaffolds combined with LIPUS stimulation were determined using a live/dead cell staining kit (Beyotime, China). The BMSCs were cultured on the scaffolds with or without LIPUS stimulation for 4 and 7 days. The medium was then removed with 200 μL of Calcein AM/PI staining solution added into every well and incubated for 1 h. The scaffolds were washed with PBS and observed under a fluorescence microscope. Bliss independence analysis was performed as


$$\text{E}=({\text{N}}_{\text{treatment}}–{\text{N}}_{\text{control}})/{\text{N}}_{\text{control}}\times 100\%.$$

The predicted additive effect was calculated as


$${\text{E}}_{\text{Bliss}}={\text{E}}_{1}+{\text{E}}_{2}-({\text{E}}_{1}\times {\text{E}}_{2})/100\%,$$

where *E*_1_ and *E*_2_ are the percentage effects of the composite scaffold and LIPUS, respectively.


$$\text{Synergy}\;\text{score}={\text{E}}_{\text{combine}}-{\text{E}}_{\text{Bliss}},$$

a positive value indicates synergy.

### Cell recruitment

The migration of BMSCs in each group was quantitatively assessed using the Transwell experiments. In brief, BMSCs were seeded into the upper chamber of Transwell inserts (24-well insert, pore size 8 μm) at the density of 3 × 10^4^, while the scaffolds were placed in the lower chamber containing 500 μL of complete culture medium. With or without LIPUS stimulation for 10 min and further incubated for 12 h, the cells migrated to the lower membrane surface were fixed using 4% paraformaldehyde (Boster, China), stained with 0.1% crystal violet and washed with double distilled water. The samples were observed under a microscope, five random fields were selected for quantifying the migrated cells using ImageJ software.

### Osteogenic differentiation

BMSCs were cultured on scaffolds with or without LIPUS stimulation in osteogenic medium consisting of DMEM high-glucose medium (Hyclone, USA), 10% FBS and 1% penicillin–streptomycin, supplemented with 10 mmol/L β-glycerophosphate, 0.1 mmol/L dexamethasone and 50 µg/mL ascorbic acid. After 7 days, alkaline phosphatase (ALP) staining was performed using an ALP kit (Beyotime, China) according to the manufacturer’s instructions. The BMSCs on scaffolds were washed with PBS, fixed with 4% paraformaldehyde, incubated with 200 μL of BCIP/NBT working solution at 37°C for 20 min in the dark. For mineralization assessment after 14 days of osteogenic induction, Alizarin Red S (ARS) staining was performed using an ARS kit (Beyotime, China). The samples were washed and fixed with the kit-provided fixative for 20 min, incubated with ARS solution for 30 min, then observed under a microscope.

### Cell immunofluorescence staining

To further evaluate the osteogenic and tenogenic differentiation of BMSCs based on different groups, immunofluorescence staining was performed to determine the expression of osteogenesis-related proteins of runt-related transcription factor 2 (RUNX2) and osteopontin (OPN), and the tenogenesis-related proteins of scleraxis (SCX) and tenomodulin (TNMD). After being cultured for 4 and 7 days, BMSCs on scaffolds were washed with PBS, fixed using 4% paraformaldehyde and blocked with 10% goat serum (Sigma Aldrich, USA) containing 0.2% Triton X-100 (Solarbio, China). The samples were washed again with PBS, incubated with corresponding primary antibody (rabbit monoclonal antibody, dilution ratio 1:500, Abcam, UK) at 4°C overnight. Next, the samples were washed using PBS with 0.05% Tween-20 (Solarbio, China), light-shielded incubated with secondary antibody (Alexa Fluor 488-conjugated goat anti-rabbit IgG, dilution ratio 1:500, ZSGB-BIO, China), then washed using PBST. The cell nucleus was counterstained with 4′,6-diamidino-2-phenylindole dihydrochloride (DAPI, Beyotime, China). The sample was enclosed between two coverslips and observed under a fluorescence microscope.

### Real-time quantitative polymerase chain reaction

The osteogenesis‑related (RUNX2, OPN) and tenogenesis‑related (SCX, TNMD) gene expression levels in BMSCs of different groups were analysed by RT‑qPCR. All primer sequences are listed in Supplementary Appendix Table 1. GAPDH was used as the internal reference gene. The mRNAs of BMSCs were extracted using a Total RNA Micro Kit (Megan, China). The concentration of the extracted RNA was determined using a microvolume spectrophotometer (Thermo Fisher, USA). Reverse transcription was performed using a gDNA removal cDNA synthesis kit (CWbio, China) on a PCR reverse transcription device (Bioer, China). The resulting cDNA was then mixed with UltraSYBR Mixture (CW bio, China), primers of target genes and RNase-free water. The mixture was added to a 96-well PCR plate and placed into the real-time quantitative PCR device for amplification according to the manufacturer’s protocol.

### Western blot analysis

The protein expression levels of β‑catenin and Piezo1 in BMSCs of different groups were analysed by WB. After being cultured for 4 days, the total protein was extracted from BMSCs using RIPA lysis buffer supplemented with PMSF (Beyotime, China). The protein concentration was determined using a BCA protein assay kit (Beyotime, China). Equal amounts of protein were mixed with 5× reducing protein loading buffer and denatured by boiling in water bath for 10 min. The protein samples were separated by SDS‑PAGE gel and transferred onto PVDF membranes pre-activated in methanol. The membranes were then blocked with 5% non‑fat dry milk in TBST for 1 h at room temperature. The membranes were incubated overnight at 4°C with primary antibodies. GAPDH and β‑tubulin were used as loading controls for β‑catenin and Piezo1, respectively. After washing with TBST, the membranes were incubated with HRP‑conjugated secondary antibody (1:5000) for 30 min at room temperature. After three additional washes with TBST, the membranes were incubated with ECL working solution, and the protein bands were visualized using a chemiluminescence imaging system (SCG-W3000 Plus, Servicebio, China).

### Biocompatibility evaluation of BMP-2/bFGF@GM-PLA scaffolds *in vivo*

All *in vivo* experimental procedures were reviewed and approved by the Animal Experiment Ethics Committee of Chongqing Medical University (NO.IACUC-CQMU-2024-0859). Twelve SD rats were randomly assigned to four groups: a control group and three experimental groups (*n* = 3 each) implanted with BMP‑2/bFGF@GM‑PLA scaffolds. Rats in the experimental groups received scaffolds subcutaneously under pentobarbital sodium anesthesia (50 mg/kg, Sinopharm, China) and were sacrificed at 2, 4 and 6 weeks post‑implantation, respectively. At each time point, the retrieved scaffolds were evaluated for degradation, blood samples were collected for hematological analysis and major organs (heart, liver, spleen, lung and kidney) were processed for hematoxylin–eosin (HE) histopathology. Implant‑adjacent tissue was used to measure inflammatory cytokine measurement by ELISA.

### Animal modeling and *in vivo* implantation of scaffolds

To assess the *in vivo* performance of the scaffolds, 50 male SD rats (mean mass 280 ± 20 g, age 10 weeks, Dashuo Bioengineering, China) were randomly allocated into five groups: Sham group, PLA group, BMP-2/bFGF@GM-PLA group, LIPUS + PLA group, LIPUS + BMP-2/bFGF@GM-PLA group. The contralateral shoulders of the Sham group were served as the normal group, representing the native tendon–bone connection. The rotator cuff tear model was established on the left upper extremity based on established method [25]. Rats were anesthetized with pentobarbital sodium (50 mg/kg, Sinopharm, China), a 1 cm longitudinal incision over the greater tuberosity of the humerus and blunt dissection of the deltoid muscle were made. The supraspinatus tendon insertion was exposed and sharply transected, and the cartilage in the footprint area was removed. For the surgical repair, a 2 mm bone tunnel connected to the marrow cavity was drilled through the greater tuberosity of the humerus, and the supraspinatus tendon stump was reattached to its anatomical footprint by passing the sutures through the bone tunnel to complete the transosseous repair. In each experimental group, a 5 mm × 3 mm scaffold was placed over the transected supraspinatus tendon stump at the repair site. Sutures were passed through the scaffold and the tendon, then through the bone tunnel to the greater tuberosity, securing the tendon to the bone and preventing scaffold displacement. Rats in the Sham group received only surgical repair without scaffold implantation or LIPUS treatment. After surgery, the rats were allowed to recover with normal activity in their cages and injected with antibiotics for 3 days.

### Post-operative LIPUS treatments

For the ultrasound-treated groups, 2 weeks of LIPUS treatments were initiated on the first day after the surgical repair. The rats in the ultrasound-treated groups received LIPUS irradiation on the repaired site through coupling gel. The parameters were set at an intensity of 100 mW/cm^2^, a frequency of 1 MHz and a duty cycle of 20% for 20 min daily.

### Biomechanical test

Rats were sacrificed at 6 weeks postoperatively. The complex of supraspinatus tendon and proximal humerus was harvested. For the biomechanical test, all surrounding tissues except for the supraspinatus and grafted scaffold were carefully removed. The fresh specimens (*n* = 5 in each group) were tested using an electronic mechanical testing machine (UniVert, CellScale, Canada), with the scapular end and humeral end placed in the fixture and secured with screws to prevent slipping from the apparatus. The specimen was stretched at rate of 10 mm/min by applying a tensile force along the axis of the tendon until failure. The tensile load and corresponding displacement were recorded, the maximal failure load was measured. The slope of the linear region of the load–deformation curve was taken as the stiffness.

### Micro-computed tomography imaging

The specimens (*n* = 5 in each group) were fixed in 4% paraformaldehyde for 24 h then underwent micro-CT scanning (NMC-200, Pingsheng Scientific, China). Scanning parameters were set as follows: tube voltage: 80 kV, tube current: 0.06 mA, voxel size: 35 μm, spatial resolution: <7.5 μm. The image slices were reconstructed using N-Recon software. A 5 mm × 3 mm rectangular region of interest was selected centered on the insertion of the supraspinatus tendon to include the humeral footprint and the bone tunnel. The presence of heterotopic ossification was observed, the bone volume/tissue volume (BV/TV), trabecular thickness (Tb.Th) and bone mineral density (BMD) were quantitatively evaluated.

### High-frequency ultrasound imaging

High-frequency ultrasound scanning was performed to observe the general condition of repaired tendon at 6 weeks postoperatively before euthanasia of the rats. The target shoulder was shaved and exposed in a lateral recumbent position for examination. The parameters of ultrasound (Aplio 800, Japan) were set as follows: frequency range: 8–24 MHz, default depth: 1.5 cm, default gain: 100, dynamic range: 65, frame rate: 35/s, minimum focal depth: 0.7 mm, minimum resolution: 32 μm. A high-frequency probe (L24) was used to locate the greater tubercle of humerus and scapula to identify the supraspinatus tendon area. The thickness and echogenicity of the area were observed.

### Histological analysis

Histological evaluation was performed after micro-CT examination. The specimens were decalcified for 3 weeks, dehydrated through graded ethanol, then embedded in paraffin. About 5 μm-thick tissue sections containing the coronal plane of the TBI and the greater tuberosity were stained with HE, safranin O-fast green (SO-FG), Masson trichrome (MT) and Picrosirius red (PSR). HE staining was used to observe the overall morphology of TBI, SO-FG staining was used to evaluate the fibrocartilage regeneration, MT staining was used to assess the accumulation and arrangement of collagen fibers, PSR staining was used to observe the different types of collagen fibers under polarized light. Semiquantitative analysis was performed using a histological scoring system (Supplementary Appendix Table 2) according to existing methods [26].

### Histological immunofluorescence staining

About 5 μm-thick tissue sections were deparaffinized, rehydrated, and then subjected to antigen retrieval in 0.1 mol/L citrate buffer (pH 6.0) heated by microwave. After cooling, the sections were rinsed in PBS, circled around the tissue with a histology marker and blocked with 3% BSA. The samples were incubated overnight at 4°C with primary antibodies, including rabbit anti-OPN (dilution ratio 1:500, servicebio, China), anti-RUNX2, anti-inducible nitric oxide synthase (iNOS) and anti-CD163 (dilution ratio 1:200, servicebio, China). Following PBS washes, CY3-conjugated goat anti-rabbit secondary antibodies were light-shielded incubated for 1 h. Sections were counter-stained with DAPI for 10 min, treated with autofluorescence quencher solution for 5 min and then mounted with an anti-fade medium.

### Statistical analysis

Statistical analyses were performed using GraphPad Prism version 10.3. Data were presented as mean ± SD. Comparisons between two groups were performed using independent-samples *t*-test for normally distributed and homoscedastic data; Welch’s *t*-test was applied when variances were unequal. For multiple-group comparisons, one-way analysis of variance (ANOVA) was used, followed by Tukey’s or Šídák’s *post hoc* pairwise comparisons. Statistical significance was set at *P* < 0.05.

## Results and discussion

### Characterization of BMP-2/bFGF@GM-PLA scaffolds

**Figure 1:**
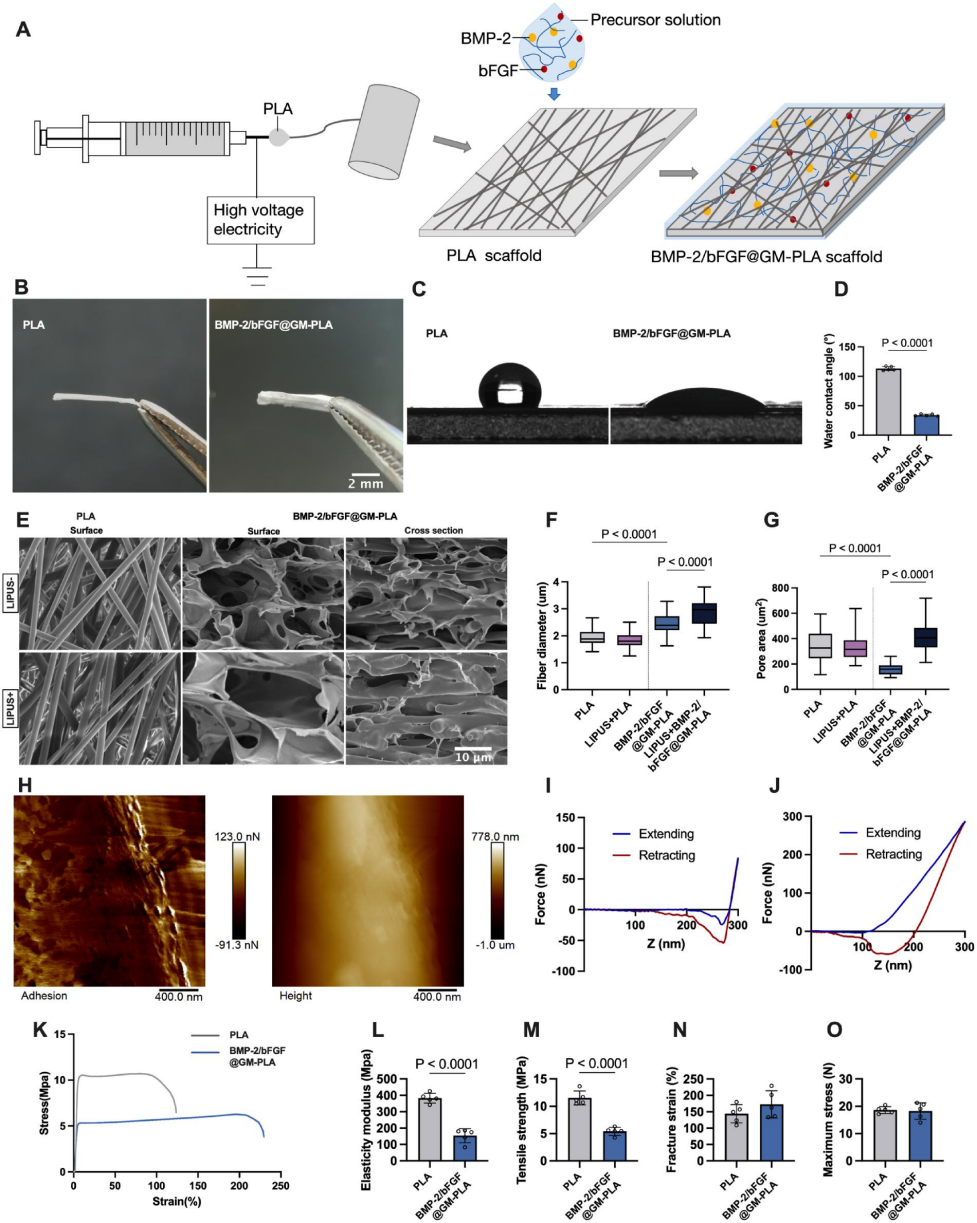
Characterization of the BMP-2/bFGF@GM-PLA scaffolds. (**A**) Schematic diagram of the preparation process of PLA and BMP-2/bFGF@GM-PLA scaffolds. (**B**) Appearance of PLA and BMP-2/bFGF@GM-PLA scaffolds. (**C** and **D**) Representative images of the water contact angle measurement on the scaffolds and the quantitative analysis (*n* = 5). (**E**) Representative SEM images revealing morphology of PLA and BMP-2/bFGF@GM-PLA scaffolds and the effects of LIPUS processing (scale bar: 10 μm). (**F** and **G**) Quantitative analysis of morphological characters (**F**: fiber diameter, *n* = 50; **G**: pore area, *n* = 20). (**H**) Representative AFM images revealing adhesion and topography of the BMP-2/bFGF@GM-PLA scaffolds with LIPUS stimulation. (**I** and **J**) Representative AFM force–distance curves obtained from the hydrogel-coated PLA fiber region and the fiber-free hydrogel region of the BMP-2/bFGF@GM-PLA scaffolds. (**K**) Representative stress–strain curves obtained for the scaffolds. (**L–O**) Mechanical property analysis of the scaffolds (**L**: elastic modulus; **M**: tensile strength; **N**: fracture strain; **O**: maximum stress. *n* = 5). (For panels **D**, **F**, **G**, **L**–**O**, data are expressed as means ± SD, only statistically significant *P*-value is displayed).

The composite scaffolds of BMP-2/bFGF@GM-PLA were fabricated through incorporation of BMP-2 and bFGF loaded GM hydrogel within the PLA electrospun mat (Figure 1A). The photographic images of PLA and BMP-2/bFGF@GM-PLA samples are presented in Figure 1B. The hydrogel precursor infiltrated the porous PLA mat by capillary action and was subsequently immobilized by photopolymerization, leading to physical integration between the hydrogel and the PLA fibers. PLA contains hydrophobic groups within its structure. In contrast, the GM hydrogel comprises polymer chains crosslinked into a 3D network structure that closely mimics the natural ECM, thereby exhibiting low immunogenicity and high hydrophilicity [27]. Our results showed the average water contact angle of the PLA fibers was 113.2°, whereas it was significantly reduced in the BMP-2/bFGF@GM-PLA scaffolds to 34.1° (Figure 1C and D). This significant decrease indicated that the incorporation of the hydrogel significantly enhanced the hydrophilicity of the PLA fibers, thereby endowing the composite scaffolds with better biocompatibility.

SEM images revealed that both scaffolds had porous structures (Figure 1E). Porosity plays a critical role in cell adhesion, infiltration and nutrient exchange within scaffolds [28]. The fibers of the PLA scaffold exhibited smooth surfaces with an average fiber diameter of 1.9 μm and an average pore area of 338.8 μm^2^ (Figure 1F and G). The BMP-2/bFGF@GM-PLA scaffolds exhibited hydrogel encapsulation around PLA fibers on SEM, the average diameter significantly increased to 2.4 μm, indicating that the hydrogel precursor successfully infiltrated the PLA fiber network and was immobilized after photo-crosslinking. After LIPUS intervention, the fiber size and pore area of PLA scaffolds remained unchanged, whereas those of the BMP-2/bFGF@GM-PLA scaffolds increased significantly. SEM further confirmed that the hydrogel remained intimately wrapped around the PLA fibers without visible detachment.

The surface adhesion of the composite scaffolds after LIPUS treatment was further evaluated under AFM (Figure 1H). The force–distance curves showed that the fiber-free hydrogel region exhibited deeper indentation and smoother retraction profiles (Figure 1I), consistent with a softer hydrogel-dominated response. In contrast, the hydrogel-incorporated PLA fiber region showed shallower indentation and a more complex retraction behavior (Figure 1J), indicating greater local mechanical resistance due to the underlying fiber support. The similar pull-off forces observed in the two regions suggested comparable apparent adhesion that primarily governed by the hydrogel surface (Supplementary Figure S1). Together, the AFM and SEM results indicated that LIPUS exposure did not cause detectable interfacial delamination or loss of hydrogel coverage from the fiber surface, thereby supporting the structural stability of the composite scaffolds after LIPUS exposure.

Rotator cuff scaffolds need to possess mechanical properties comparable to those of TBI. In this study, both scaffolds presented good flexibility for use as rotator cuff patches, and exhibited characteristic stress–strain curves, featuring an initial high-modulus linear elastic phase followed by a post-yield region marking fiber structural failure (Figure 1K). These curves qualitatively match the native tendon stress–strain response: a brief low-stiffness toe region, a linear high-modulus phase, then a yield plateau with progressive strain-softening due to accumulating micro-damage of tendon, and abrupt ultimate failure [29]. The elastic modulus and tensile strength of the BMP-2/bFGF@GM-PLA scaffolds were 153.4 ± 42.8, 5.4 ± 0.7 MPa, respectively, which were lower than those of the PLA scaffolds (Figure 1L–O), suggesting enhanced softness and diminished tensile strength of the composite scaffolds following incorporation with GM. Nevertheless, the BMP-2/bFGF@GM-PLA scaffolds maintained the maximum stress and fracture strain values comparable to those of the PLA scaffolds, as the PLA fibers served as the skeleton of the composite scaffolds. These findings suggested that the composite scaffold could provide adequate mechanical support for rotator cuff applications, outperforming pure hydrogel scaffolds in this regard [30].

**Figure 2:**
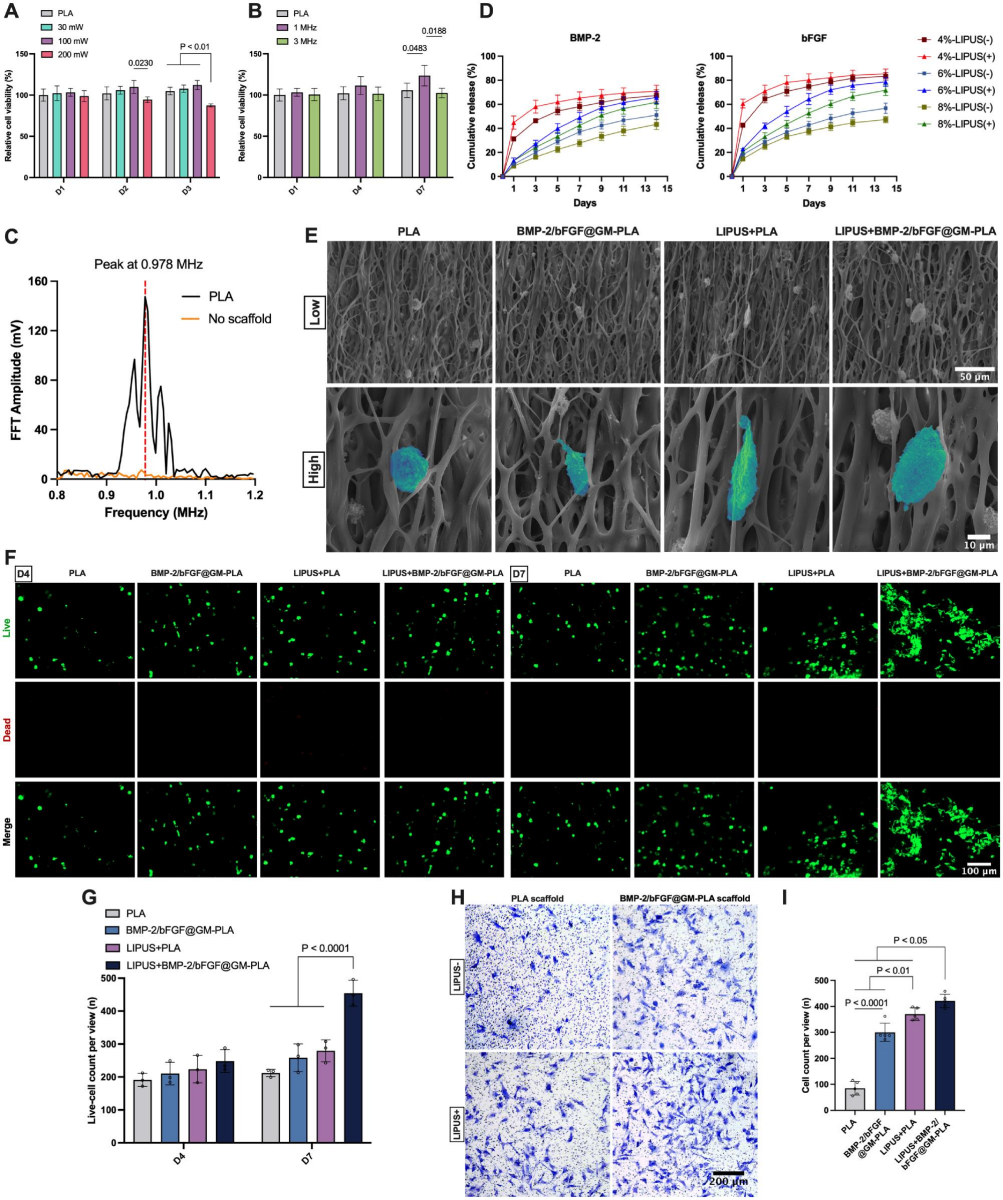
LIPUS stimulated responsiveness of BMP-2/bFGF@GM-PLA system and *in vitro* cellular function. (**A** and **B**) CCK8 result of BMSCs on PLA scaffolds treated with LIPUS at different parameters (**A**: intensities; **B**: frequencies. *n* = 3). (**C**) Representative FFT spectra of PLA scaffold vibration and control under LIPUS stimulation. (**D**) Cumulative release curves of BMP-2 and bFGF from the composite scaffolds of GM at different concentrations with or without LIPUS stimulation (*n* = 3). (**E**) Representative SEM images of BMSCs cultured on PLA and BMP-2/bFGF@GM-PLA scaffolds with or without LIPUS stimulation at 7 days (low magnification, scale bar: 50 μm; high magnification, scale bar: 10 μm). (**F**) Living-dead staining images of BMSCs cultured on scaffolds with or without LIPUS stimulation at 4 days and 7 days (scale bar: 100 μm). (**G**) Quantitative analysis of live cell count (*n* = 3). (**H**) Images of migrated BMSCs in transwell assays (scale bar: 200 μm). (**I**) Quantitative analysis of the migrated cell counts (*n* = 5). (For panels **A**, **B**, **D**, **G** and **I**, data are expressed as means ± SD, one-way ANOVA, only statistically significant *P*-value is displayed).

To optimize the acoustic responsiveness of PLA scaffolds, a set of LIPUS parameters was evaluated. While a slight increase in cell viability was observed at 30 and 100 mW/cm^2^ intensities compared to those without LIPUS stimulation, BMSCs on PLA scaffolds exposed to 200 mW/cm^2^ exhibited apparent damages to viability from day 2 (Figure 2A), indicating that excessive acoustic energy elicited pronounced cytotoxicity. For frequency, BMSCs cultured on PLA scaffolds and exposed to 1 MHz LIPUS for 7 days exhibited a significant increase in viability compared to those without LIPUS stimulation, whereas 3 MHz produced no beneficial effect (Figure 2B), indicating better acousto-mechanical responsiveness of the PLA scaffold under 1 MHz. Consequently, all subsequent *in vitro* and *in vivo* experiments were conducted using LIPUS configured at an intensity of 100 mW/cm^2^ and a frequency of 1 MHz, with a 20% duty cycle.

An intensity of 100 mW/cm^2^ at 1 MHz has been validated to promote osteoblast proliferation and osteogenic gene expression through a LIPUS-responsive scaffold [24]. This intensity is also widely used in mechanotransduction studies, with a minimal temperature rise to ensure non-thermal mechanical effects [15]. A 20% duty cycle was chosen, as it upregulates osteogenic genes more pronouncedly than 50%, suggesting superior osteogenic induction at a lower duty ratio [14]. Collectively, these parameters are well-aligned with both clinical applications of tissue repair [31] and basic science research, supporting the clinical relevance and translational potential of the approach.

LDV measurements revealed that the PLA scaffold responded to LIPUS with a distinct vibration peak at 0.97 ± 0.02 MHz, closely matching the nominal driving frequency of 1.0 MHz. In contrast, the control condition without the scaffold did not exhibit a comparable peak at this frequency (Figure 2C), indicating that the detected signal originated from the scaffold response than from background artifacts. Quantitative analysis showed a scaffold vibration with a peak-to-peak displacement of 1.70 ± 0.61 nm and a derived velocity of 5.18 ± 1.92 mm/s. These values were consistent with a high-frequency oscillatory system, where a nanoscale displacement yields a measurable velocity. This nanoscale displacement should be interpreted as a spatially averaged scaffold-level response, reflecting the collective motion within the laser spot area that covered multiple fibers and pores rather than the displacement of a single fiber. Collectively, the LDV results indicated that the PLA scaffold mechanically responded to LIPUS at the driving frequency and provided a physical basis for ultrasound-induced mechanotransduction.

### LIPUS-stimulated drug release from BMP-2/bFGF@GM-PLA scaffolds *in vitro*

The composite scaffolds of GM at concentration of 4%, 6% and 8% (w/v) demonstrated sustained release of the growth factors. The 4% concentration scaffold exhibited rapid release of BMP-2 and bFGF, while the 6% and 8% concentration scaffolds exhibited more controlled releases (Figure 2D). The release kinetics indicated that the lower crosslinking densities in GM scaffolds, due to their looser network structure, facilitate more rapid initial release of growth factors through enhanced diffusion, while higher densities provide more sustained delivery. When subjected to LIPUS stimulation, scaffolds of different concentrations all exhibited higher cumulative release of BMP-2 and bFGF compared to those without LIPUS stimulation, while the 4% concentration group showed an undesired burst release under LIPUS. Subsequent composite scaffolds were thereby fabricated using GM of 6% concentration. The weight loss rate of BMP-2/bFGF@GM-PLA scaffolds significantly increased with LIPUS stimulation (Supplementary Figure S2). Given the SEM results showing that LIPUS caused morphological changes in the GM hydrogel while preserving the PLA fibrous structure, it was reasonable to infer that LIPUS stimulation could accelerate the degradation of GM, thereby facilitating the diffusion of growth factor molecules from the composite scaffolds.

### Biocompatibility, cell recruitment and differentiation potential of the BMP-2/bFGF@GM-PLA scaffolds under LIPUS stimulation *in vitro*

#### Biocompatibility and cell proliferation

Biocompatibility is a fundamental requirement for scaffolds and external stimuli. Given that PLA is commonly used as commercial rotator cuff scaffold in clinic settings, the PLA group served as the control in this study. SEM images of BMSCs cultured on PLA and BMP-2/bFGF@GM-PLA scaffolds with or without LIPUS stimulation for 7 days revealed cell attachment and infiltration through the porous scaffold structures. The cells exhibited larger volumes and elongated morphology on scaffolds subjected to LIPUS stimulation compared to those without ultrasonic processing (Figure 2E), implying that LIPUS facilitated cell adhesion and enhanced spreading on scaffolds. Live/dead staining of BMSCs cultured on scaffolds for 4 and 7 days consistently exhibited green fluorescence, with minimal red fluorescence from dead cells observed (Figure 2F), verifying the biological safety of both the scaffolds and LIPUS stimulation. This aligned with the common view that PLA and GM materials, as well as LIPUS stimulation, are biocompatible.

The quantity of live cells significantly increased in the LIPUS +BMP-2/bFGF@GM-PLA group after 7 days of culture (Figure 2G), indicating a promoting effect on cell proliferation of LIPUS-stimulated BMP-2/bFGF@GM-PLA system. Bliss independence analysis revealed that the combined effect of the composite scaffold and LIPUS was 114.3%, while the predicted additive effect was 46.8%. The synergy score was 67.5% (95% CI: 53.5–81.5%), demonstrating a significant synergistic effect of the combined system in promoting cell proliferation.

#### Cell recruitment

The migration of stem cells to the tissue defect site and onto the scaffold is a prerequisite for subsequent cell proliferation and differentiation to achieve a reparative effect. Transwell assay (Figure 2H and I) showed that the quantity of migrated cells was higher in the LIPUS + PLA group than in the PLA group, indicating that the application of LIPUS to the PLA scaffolds promoted cell migration. This effect was presumably related to the mechanical vibrations in the PLA fibers induced by LIPUS stimulation, a finding consistent with previous research showing that LIPUS led to increased cell recruitment on 3D-printed PLA scaffolds [19]. Besides, the BMP-2/bFGF@GM-PLA groups exhibited a better promoting effect on cell migration compared to the PLA groups. This enhancement was expected to result from the controlled release of BMP-2 and bFGF, which functioned as chemotactic factors. BMP-2 is reported to increase the migration of BMSCs *in vitro* via activation of the CDC42/PAK1/LIMK1 pathway, and bFGF is shown to enhance cell migration by activation of the HK2/β-catenin pathway [32, 33]. In particular, the LIPUS + BMP-2/bFGF@GM-PLA group exhibited the highest migrated cell counts among all groups, indicating that the LIPUS-stimulated BMP-2/bFGF@GM-PLA system effectively integrated both mechanical and chemical cues, resulting in the optimal cell recruitment.

#### Osteogenic and tenogenic differentiation potential

**Figure 3:**
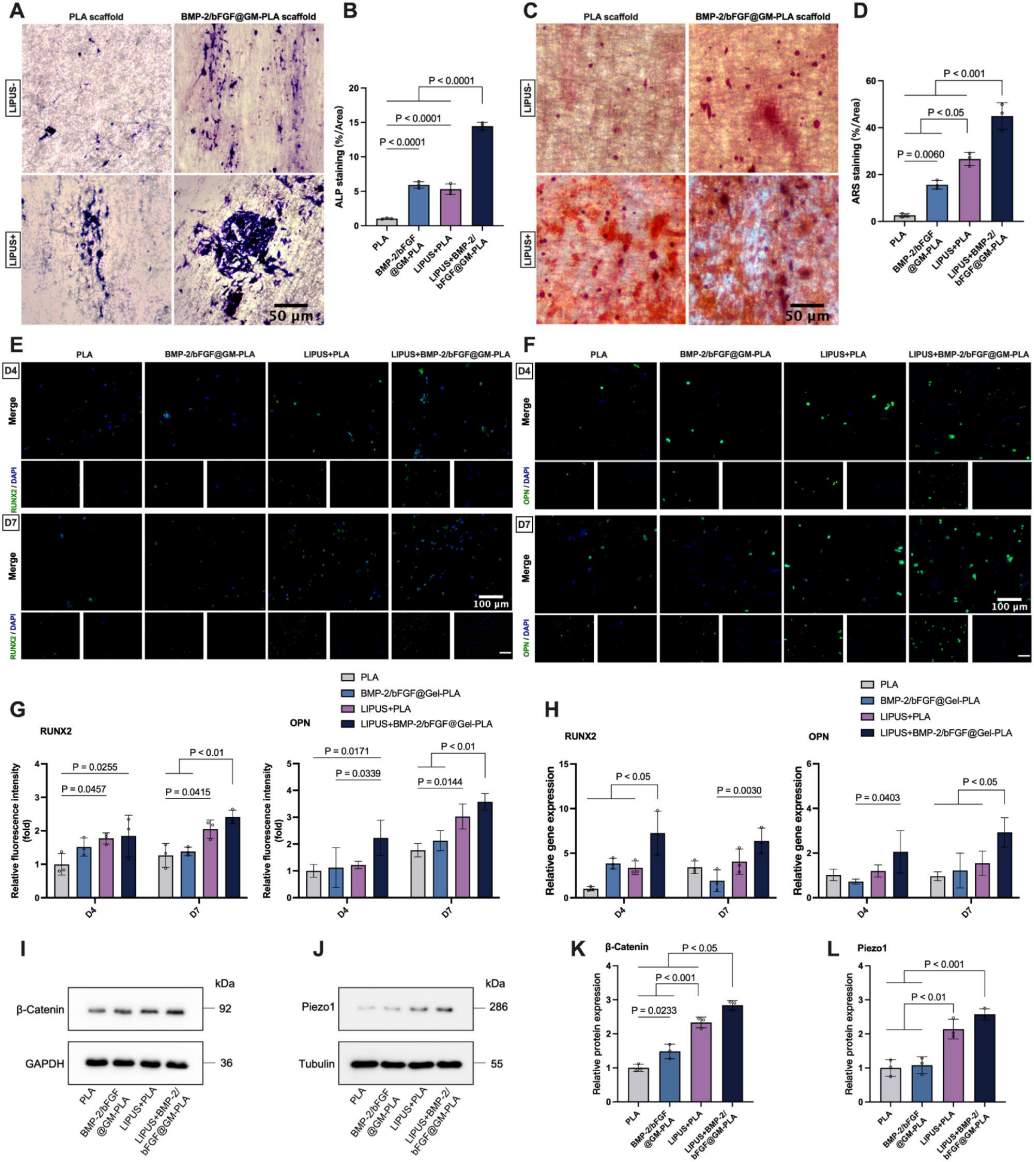
LIPUS stimulated BMP-2/bFGF@GM-PLA scaffolds promoted the osteogenic differentiation *in vitro*. (**A**) The alkaline phosphatase (ALP) staining of BMSCs on scaffolds with or without LIPUS stimulation at 7 days. (**B**) Quantitative analysis of ALP staining. (**C**) The Alizarin Red S (ARS) staining of BMSCs on scaffolds with or without LIPUS stimulation at 14 days (scale bar: 50 μm). (**D**) Quantitative analysis of ARS staining. (**E** and **F**) Immunofluorescence staining of biomarkers of the BMSCs osteogenesis on the PLA and BMP-2/bFGF@GM-PLA scaffolds with or without LIPUS stimulation on days 4 and 7 (scale bar: 100 μm. **E**: RUNX-2; **F**: OPN). (**G**) Analysis of fluorescence intensity of RUNX-2 and OPN of different groups at 4 and 7 days. (**H**) The relative mRNA level of RUNX-2 and OPN of different groups at 4 and 7 days. (**I** and **J**) Western blot analysis of β‑catenin (**I**) and Piezo1 (**J**) expression of different groups. (**K** and **L**) Quantitative analysis of the protein expression of β‑catenin and Piezo1. (For panels **B**, **D**, **G**, **H**, **K** and **L** = 3, data are expressed as means ± SD, one-way ANOVA, only statistically significant *P*-value is displayed).

Enhancing rotator cuff healing requires addressing both osseous and tendinous repair. The BMP-2/bFGF@GM-PLA and LIPUS + PLA groups displayed larger ALP-positive areas at day 7, with the LIPUS + BMP-2/bFGF@GM-PLA group showing the largest area among all tested groups (Figure 3A and B), signifying elevated early-stage osteogenic differentiation activity. After 14 days of osteogenic induction, the ARS-positive areas increased sequentially across the BMP-2/bFGF@GM-PLA, LIPUS + PLA, and LIPUS + BMP-2/bFGF@GM-PLA groups (Figure 3C and D), suggesting greater calcium deposition by the BMSCs. These results indicated that the LIPUS‑stimulated BMP‑2/bFGF@GM‑PLA system consistently promoted the osteogenic differentiation of BMSCs. Notably, the combined system led to a markedly greater increase in ALP and ARS staining areas than either individual approach, indicating a synergistic effect on osteogenesis.

Immunofluorescence staining and RT-qPCR were further utilized to assess the effects of this combined system on the osteogenic differentiation of BMSCs. RUNX2, a key transcriptional factor in the early stage of osteoblastic differentiation, is involved in activating the expression of various osteogenesis-related genes, promoting the synthesis of bone matrix and participating in the formation and maturation of bone tissue [34]. OPN is continuously expressed during the process of osteoblastic differentiation, which plays a vital role in the bone formation, remodeling and mineralization [35]. After culturing BMSCs on scaffolds, the expression levels of RUNX2 and OPN in the LIPUS + BMP-2/bFGF@GM-PLA group were notably elevated compared to those of the rest groups at D4 and D7 timepoints (Figure 3E–H). Compared with LIPUS, PLA scaffolds or BMP‑2/bFGF‑loaded composite scaffolds, the LIPUS‑stimulated BMP‑2/bFGF@GM‑PLA system demonstrated clear advantages in promoting BMSC osteogenic differentiation. This dual‑cue strategy has been shown to integrate mechanical vibrations with biochemical signals, achieving a synergistic effect that single‑factor approaches cannot attain.

Western blot analysis showed that β-catenin expression was increased in the BMP‑2/bFGF@GM‑PLA group and the LIPUS + PLA group, with the highest level observed in the LIPUS + BMP‑2/bFGF@GM‑PLA group (Figure 3I and K). As a key transcriptional co‑activator in the canonical Wnt pathway, β‑catenin has been reported to promote RUNX2 expression and subsequently upregulate osteogenic markers such as OPN and ALP, while also driving cell cycle progression [36]. Thus, both LIPUS and the BMP‑2/bFGF@GM‑PLA scaffolds may enhance the proliferation and osteogenic differentiation of BMSCs by upregulating β‑catenin level. More importantly, the marked increase of β‑catenin in the combination group was consistent with the enhanced proliferation and ARS staining areas, suggesting that LIPUS and BMP‑2 may act coordinately through β‑catenin-associated signaling, which may contribute to the observed synergistic effect on proliferation and osteogenic differentiation.

In contrast, the Piezo1 expression showed no significant difference between the PLA and BMP‑2/bFGF@GM‑PLA groups, whereas it was clearly upregulated in the LIPUS + PLA and LIPUS + BMP‑2/bFGF@GM‑PLA groups (Figure 3J and L). The results suggested that Piezo1 expression was mainly responsive to LIPUS stimulation, while BMP‑2 alone exerted limited effects on this mechanosensor. Piezo1 is a mechanosensitive ion channel that transduces mechanical stimuli into intracellular Ca^2+^ signals [37]. Previous studies have also shown that mechanical or vibrational stimuli can activate mechanoreceptors such as Piezo1/2 and induce calcium influx, which enhance Wnt/β-catenin signaling and osteogenic differentiation of MSCs [38, 39]. Together, these findings suggested that Piezo1 may be involved in LIPUS-induced mechanotransduction and subsequent activation of β-catenin-related osteogenic signaling. However, how Piezo1 regulates β-catenin in this system, and the precise molecular intermediates by which BMP‑2 upregulates β‑catenin level remain to be elucidated.

**Figure 4:**
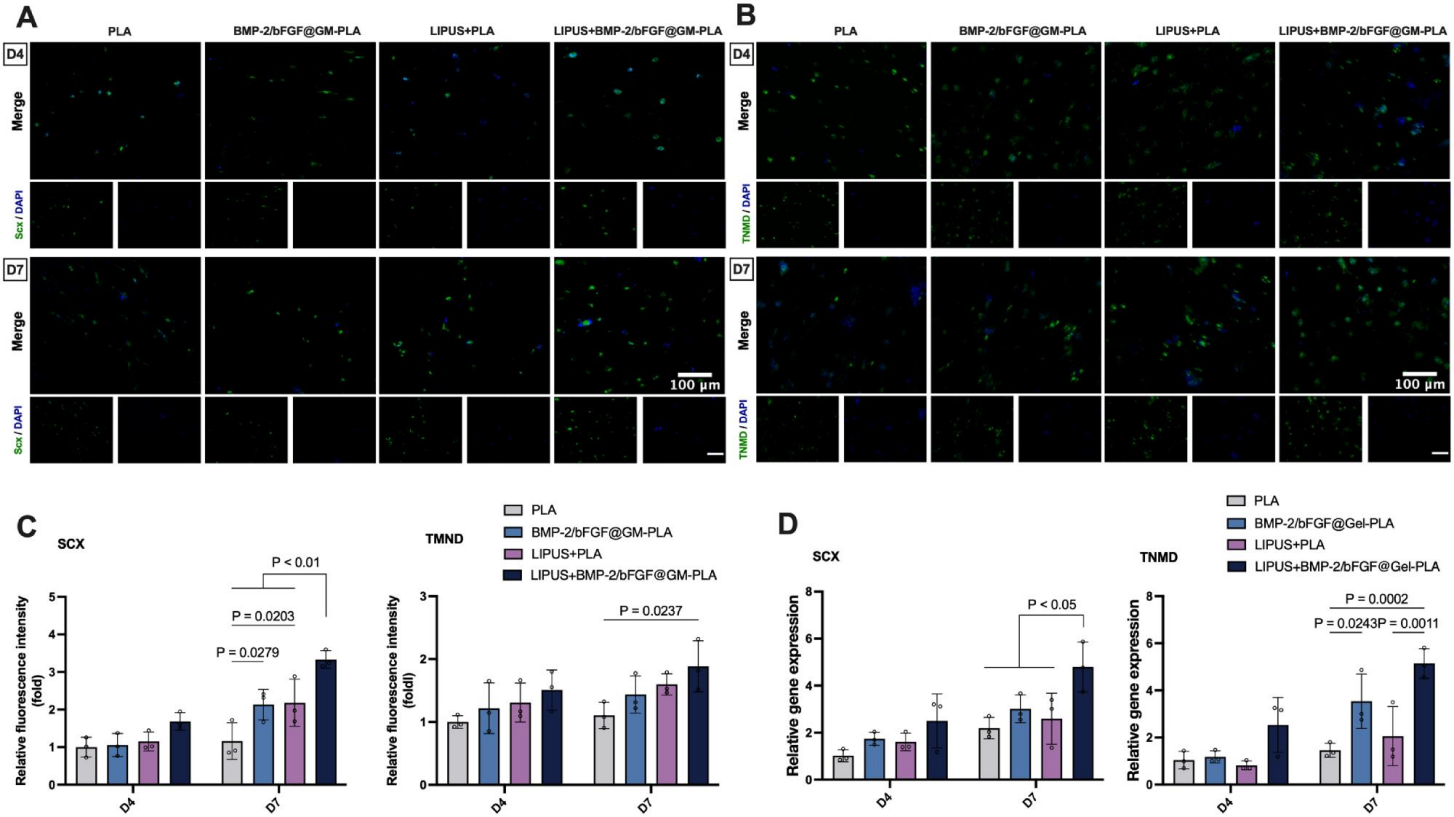
LIPUS stimulated BMP-2/bFGF@GM-PLA scaffolds enhanced the tenogenic differentiation *in vitro*. (**A** and **B**) Immunofluorescence staining of biomarkers of the BMSCs tenogenic differentiation on scaffolds with or without LIPUS stimulation on days 4 and 7 (scale bar: 100 μm. **A**: SCX; **B**: TNMD). (**C**) Analysis of fluorescence intensity of SCX and TNMD of different groups at 4 and 7 days. (**D**) The relative mRNA level of SCX and TNMD of different groups at 4 and 7 days. (For panels **C** and **D**, *n* = 3, data are expressed as means ± SD, one-way ANOVA, only statistically significant *P*-value is displayed).

In addition to osteogenesis, the system also promoted tenogenic differentiation. SCX functions as a pivotal transcriptional regulator highly expressed in the early stage of tendon development and injury repair. It promotes the expression of tendon-specific genes, such as collagen, tenascin-C and TNMD [40]. TNMD serves as a specific marker for tendon development and differentiation, with its expression closely associated with tendon cell maturation [41]. In this study, the fluorescence intensity of SCX and TNMD in the LIPUS + BMP-2/bFGF@GM-PLA group was markedly higher than in other groups at D7 timepoint, indicative of enhanced tenogenic differentiation (Figure 4A–C). Further RT-qPCR data were consistent with the immunofluorescence staining results (Figure 4D). The enhanced tenogenic differentiation of BMSCs may be attributed to the chemical cues provided by bFGF. Consistently, bFGF‑loaded nanofiber membranes have been reported to upregulate tenogenic gene expression and promote the synthesis of tendon‑specific proteins [42]. The LIPUS‑stimulated BMP‑2/bFGF@GM‑PLA system more effectively promoted both osteogenic and tenogenic differentiation of BMSCs through the integration of mechanical and biochemical cues. This combined system demonstrated significant advantages over single‑factor strategies in addressing the major challenge of simultaneous osseous and tendinous healing in rotator cuff tears.

### Effects of the LIPUS combined BMP-2/bFGF@GM-PLA system on TBI healing of rotator cuff tears *in vivo*

#### *In vivo* biodegradation and biosafety of BMP‑2/bFGF@GM‑PLA scaffolds

The *in vivo* biodegradation of the composite scaffolds was assessed by subcutaneous implantation in SD rats (Supplementary Figure S3A–C). The results indicated no death or weight loss in rat. Gross observation and the weight loss rate curve of the retrieved composite scaffolds revealed gradual partial degradation at weeks 2, 4 and 6. Routine blood and biochemical analysis at each time point revealed no abnormalities (Supplementary Figure S4), indicating that the composite scaffolds were not associated with systemic inflammation or abnormal liver, kidney or hematopoietic function *in vivo*. HE staining of major organs also showed no detectable pathological changes (Supplementary Figure S5). Moreover, inflammatory cytokines of TNF‑α and IL‑6 in the surrounding tissues did not significantly change at any time point from the control group (Supplementary Figure S6). The results collectively demonstrated that the BMP‑2/bFGF@GM‑PLA scaffolds possessed good *in vivo* biocompatibility without systemic toxicity or obvious local inflammation. The results were consistent with previous studies that PLA and GM exhibit good biodegradation and biocompatibility *in vivo* [43, 44].

#### Biomechanical properties

**Figure 5:**
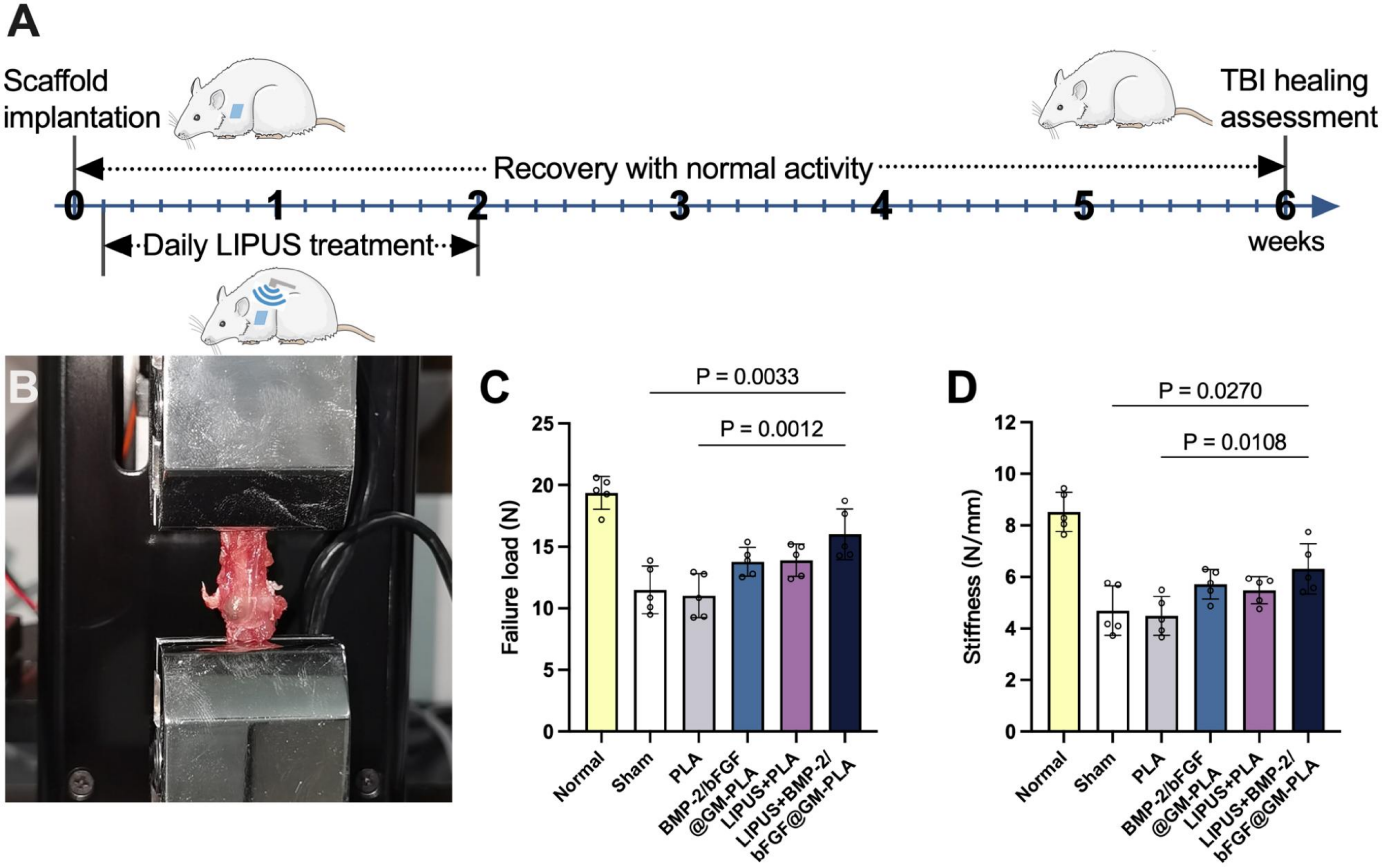
*In vivo* treatment schedule and mechanical effects of scaffold implantation combined with postoperative LIPUS for rotator cuff healing. (**A**) Treatment schedule. (**B**) Biomechanical test for tendon–bone junction stretching at 6 weeks postoperatively. (**C** and **D**) Analysis of biomechanical properties (**C**: failure load; **D**: stiffness. For panels **C** and **D**, *n* = 5, data are expressed as means ± SD, one-way ANOVA, only statistically significant *P*-value is displayed; the normal group served as qualitative reference and was excluded from the *post hoc* pairwise comparisons).

The treatment timeline for rotator cuff-injured animals is shown in Figure 5A, and the detailed procedures in Supplementary Figure S7A–C. Evaluations were performed at 6 weeks postoperatively, matching the time point of the first comprehensive clinical assessment after surgical repair in rotator cuff patients [45]. Gross specimens of tendon–bone complex in all the groups showed no obvious re-tears. Failure load, indicating the maximum force the repaired tendon–bone insertion can withstand, and stiffness, reflecting the tissue’s resistance to elastic deformation [46], were selected as parameters for the biomechanical test (Figure 5B). The results presented that the failure load and stiffness in the LIPUS + BMP-2/bFGF@GM-PLA group were significantly higher than those in the Sham and PLA groups (Figure 5C and D), suggesting that combining BMP-2/bFGF@GM-PLA scaffolds with post-operative LIPUS treatments enhanced biomechanical properties and might reduce re-tear risk. It is noteworthy that clinical studies suggest that the use of PLA scaffolds for large and massive rotator cuff tears improves patients’ postoperative symptoms and functional scores [47]. Yet the PLA group in the present study exhibited slightly inferior mechanical performance compared with the Sham group, implying that mere implantation of a PLA scaffold served more as a mechanical gap-filler that shares part of the tensile load rather than biologically augmenting the intrinsic healing capacity of the TBI. Similarly, previous work has also demonstrated that MSCs seeded collagen sponge scaffolds failed to improve rotator cuff tendon healing [48], underscoring the biological complexity of rotator cuff regeneration and the necessity for multi-factorial strategies to achieve functionally superior healing.

#### Bone tunnel healing and tendon recovery condition

**Figure 6:**
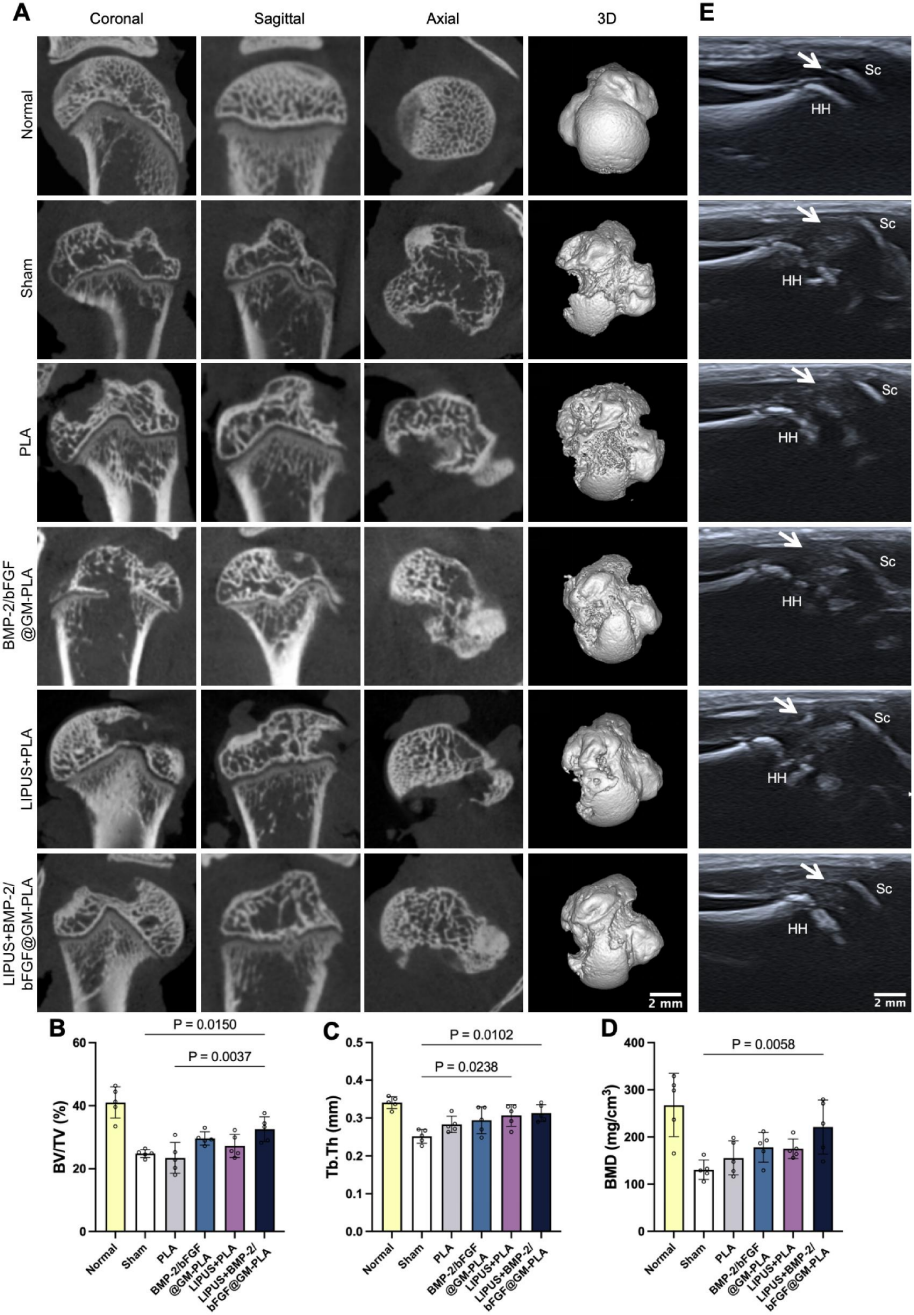
Radiologic evaluation of the bone tunnel and repaired tendon. (**A**) Representative Micro-CT and 3D reconstructed images of the humerus at 6 weeks postoperatively. (**B–D**) Analysis of the CT-based bone tissue structural parameters of the footprint and the bone tunnel area: (**B**) bone volume/tissue volume (BV/TV), (**C**) trabecular thickness (Tb.Th), (**D**) bone mineral density (BMD) (for panels **B–D**, *n* = 5, data are expressed as means ± SD, one-way ANOVA, only statistically significant *P*-value is displayed; the normal group served as qualitative reference and was excluded from the *post hoc* pairwise comparisons). (**E**) Representative high-frequency ultrasound images of the supraspinatus tendon area (HH: head of humerus, Sc: scapula, white arrow: supraspinatus tendon insertion).

The postoperative healing of the bone tunnel facilitates the stable fixation of the tendon to the bone, thereby restoring the mechanical properties and anatomic structures of TBI. Micro-CT images of the proximal humerus were used for evaluating the bone healing effects of the combined treatments of LIPUS and BMP-2/bFGF@GM-PLA. The representative images of the coronal, sagittal, axial and 3D reconstructed view of the head of humerus are presented in Figure 6A. No heterotopic ossification was observed in all the groups. The LIPUS + BMP-2/bFGF@GM-PLA group exhibited the smallest bone defect, with a significantly higher BV/TV compared to both the Sham and PLA groups. Additionally, this group demonstrated higher Tb.Th and BMD values than the Sham group (Figure 6B–D), indicating that this combined system effectively enhanced the postoperative healing of bone defects, and improved the structure of newly formed bone. For the general condition of repaired tendon, the high-frequency ultrasound images (Figure 6E) showed that the groups implanted with BMP-2/bFGF@GM-PLA scaffolds exhibited less thickening in the supraspinatus tendon area than those implanted with PLA scaffolds. This manifestation could indicate reduced edema, which was likely due to the enhanced biocompatibility of the composite scaffolds that minimized tissue irritation from the foreign implant. Among all surgical groups, the LIPUS + BMP-2/bFGF@GM-PLA group exhibited the highest degree of echo homogeneity in the supraspinatus tendon area, with a morphology most similar to that of the normal group, suggesting a favorable progression in the tendon healing process.

#### Histological structures of TBI

**Figure 7:**
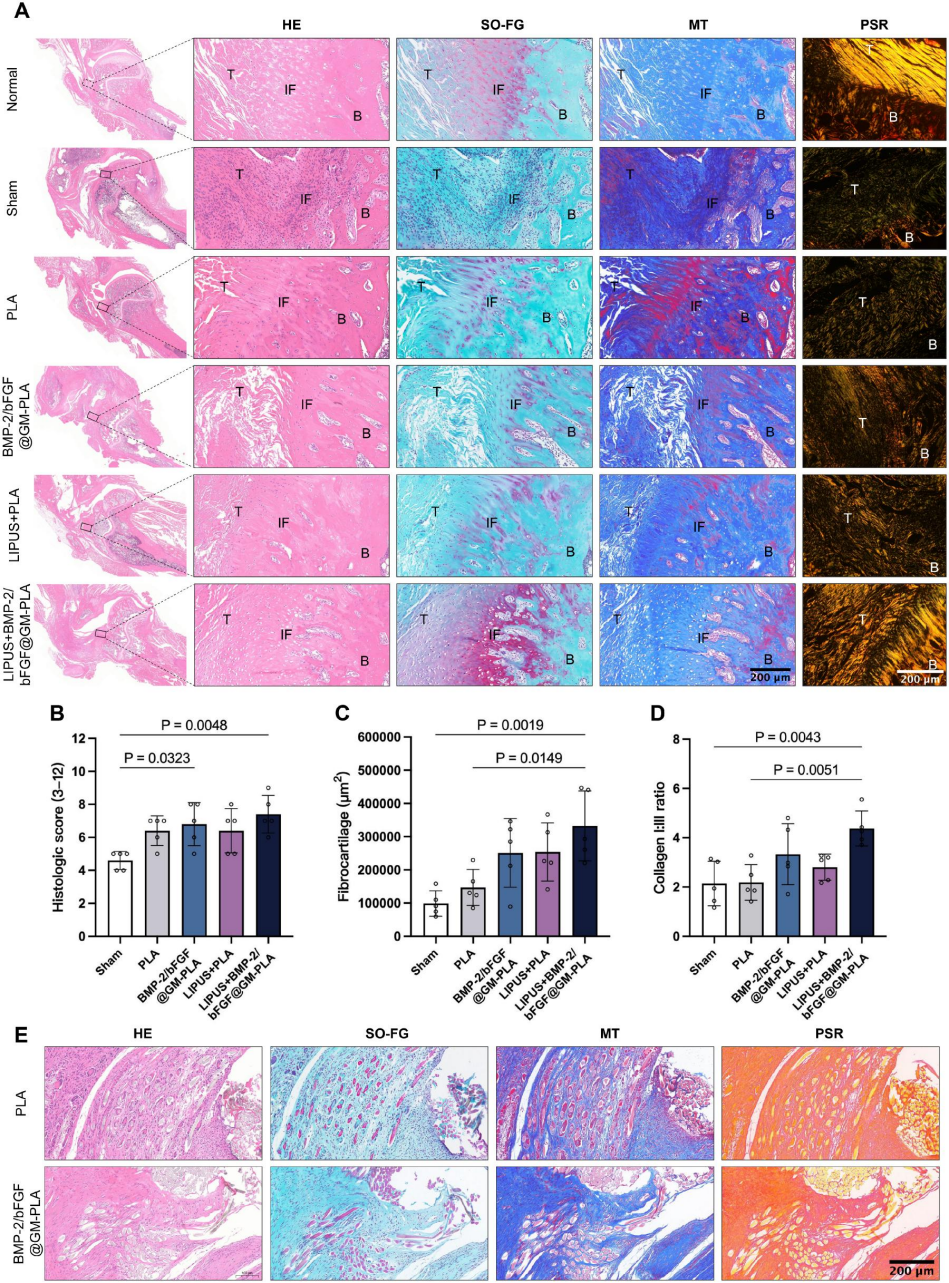
Histological evaluation of tendon–bone interface healing in rat model of rotator cuff tears. (**A**) Representative images of histological staining at the repaired site (rectangles marked the local magnified area). (**B**) Semiquantitative analysis of total histologic scores. (**C**) Analysis of newly formed fibrocartilage area on SO-FG staining. (**D**) Analysis of collagen type I:III ratio based on birefringence brightness on PSR staining. (**E**) Representative images of scaffold on histological staining. (HE: hematoxylin–eosin, MT: Masson trichrome, PSR: Picrosirius red, SO-FG: Safranin O-Fast Green, B: bone, IF: interface, T: tendon. 200×, scale bar: 100 μm. For panels **B–D**, *n* = 5, data are expressed as means ± SD, one-way ANOVA, only statistically significant *P*-value is displayed).

Histological images revealed that the interface was composed of continuous connective tissue in all groups, and no evidence of inflammatory cell infiltration. In the Sham group, the interface exhibited disordered tissue structure with the highest cell density and neovascularization, indicative of scar formation. The results showed that both PLA and BMP-2/bFGF@GM-PLA scaffolds implanted groups demonstrated improved histological structures compared to the Sham group (Figure 7A). Compared to the native interface formed by gradient regions, the interface structure in the LIPUS + BMP-2/bFGF@GM-PLA group was most distinct and closely resembling the native among all repaired groups. Significant differences in histologic scores were observed between the LIPUS + BMP-2/bFGF@GM-PLA group and the Sham group (Figure 7B). Adequate bone healing can stably anchor tendon fibers to the bone, thereby increasing the tendon–bone contact area, providing sufficient biomechanical strength with evenly distributed stress, and reducing the risk of postoperative rotator cuff re-tear [49]. HE staining revealed the structure of a typical spongy bone marrow cavity in the LIPUS + BMP-2/bFGF@GM-PLA group, and Safranin O staining showed a significantly larger area of newly formed fibrocartilage compared to the Sham and PLA groups (Figure 7C), indicating enhanced growth and remodeling of new bone and cartilage.

The formation of ordered collagen fiber bundles in the ECM also improves the biomechanical properties of the repaired interface. Type I collagen, abundant in the tendon region, is a major component of the ECM and provides high-strength mechanical support at the interface [50]. Furthermore, it plays a significant role in the mineralization process of bone tissue, acting as a nucleation site that facilitates the deposition of hydroxyapatite [51]. MT staining revealed collagen formation with more ordered fiber arrangement in the LIPUS + BMP-2/bFGF@GM-PLA group, indicating a higher degree of tendon maturation. PSR staining presented thick yellow fibers with an aligned structure, revealing of mature Type I collagen. The birefringence brightness of the collagen type I: III ratio was significantly higher compared with the Sham and PLA groups (Figure 7D), indicating enhanced generation and remodeling processes of type I collagen fibers at the repair interface, thereby facilitating TBI healing. Local observations of the undegraded fibrous scaffolds within the tissue sections revealed no significant aggregation of macrophages, neutrophils, lymphocytes or granuloma formation around the scaffold fibers. Notably, fibroblasts infiltration and collagen deposition were evident between the scaffold fibers, corroborating that the porous structure of the scaffolds was conducive to cell migration, infiltration and proliferation (Figure 7E).

#### Ossification and adverse immune reaction

**Figure 8:**
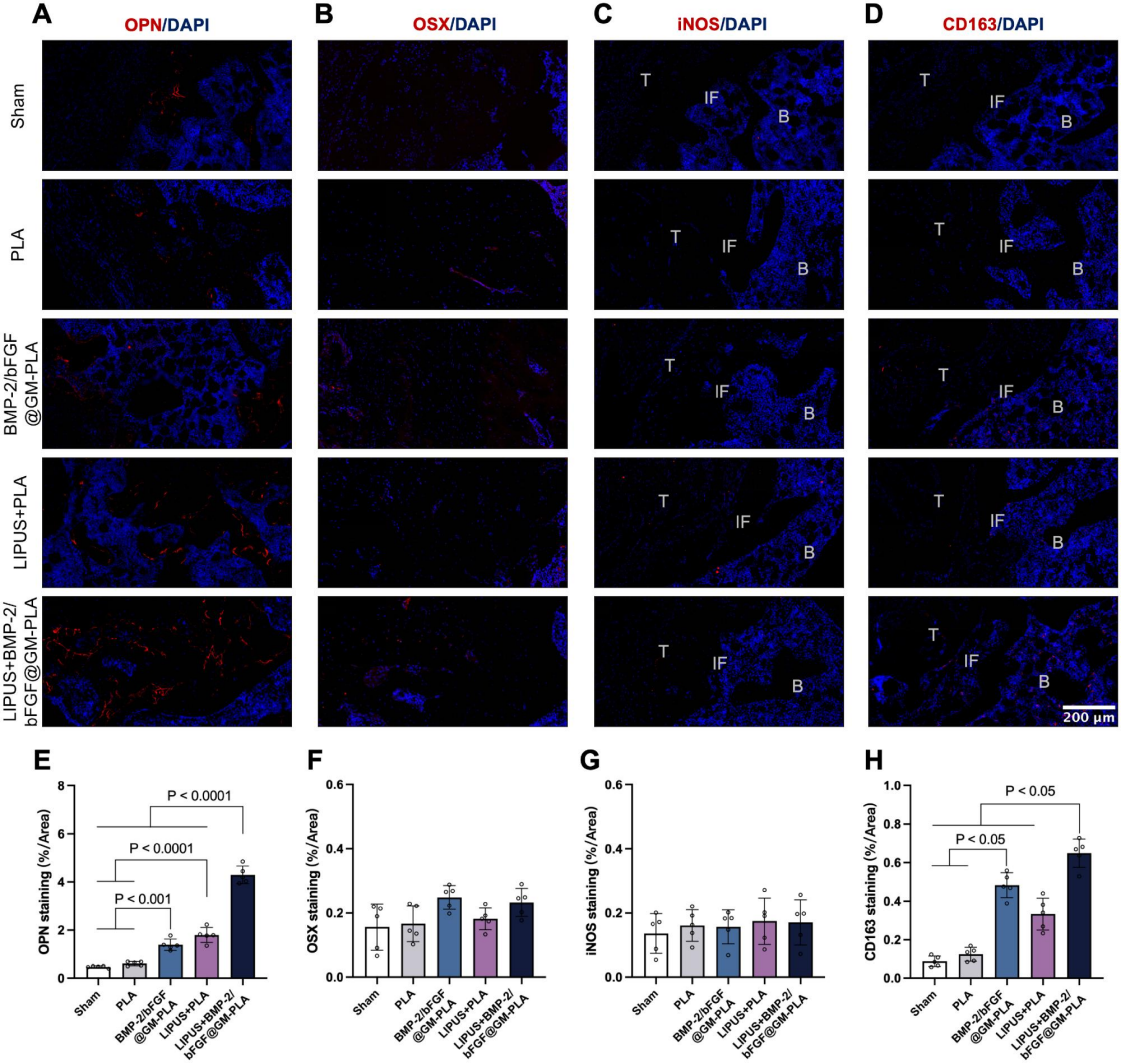
Immunofluorescence histochemistry of tendon–bone interface healing in rat model of rotator cuff tears. (**A** and **B**) Representative images of immunofluorescence staining of osteogenic biomarkers (**A**: OPN; **B**: OSX). (**C** and **D**) Representative images of immunofluorescence staining of polarization biomarkers in macrophages (**C**: iNOS; **D**: CD163). (**E**–**H**) Quantitative analysis of immunofluorescence staining (**E**: OPN; **F**: OSX; **G**: iNOS; **H**: CD 163). (B: bone, IF: interface, T: tendon, 200×, scale bar: 100 μm. For panels **E–H**, *n* = 5, data are expressed as means ± SD, one-way ANOVA, only statistically significant *P*-value is displayed).

Further histological immunofluorescence staining showed that the LIPUS + BMP-2/bFGF@GM-PLA group exhibited the largest OPN-positive area compared with other groups (Figure 8A and E). This observation corroborated the enhanced osteogenic differentiation observed *in vitro*, indicating enhanced osteogenic activity and more advanced stages of bone regeneration of the combined system. While crucial for bone regeneration, BMP-2 raises concerns regarding its potential to induce ectopic ossification, particularly at high doses. OSX is a reliable marker for identifying osteogenic activity and detecting ectopic ossification [52]. Immunofluorescence staining for OSX revealed only a slight, nonsignificant increase in the tendon region of the BMP-2/bFGF@GM-PLA scaffolds implanted groups (Figure 8B and F). This finding, corroborated by micro-CT results showing no heterotopic ossification in any group, suggested that the relatively low BMP‑2 concentration employed in the composite scaffolds did not induce significant off‑target bone formation. A key advantage of this dual‑cue strategy is that the synergistic interaction between LIPUS and BMP‑2 enhances osteogenic efficacy at a lower growth factor dose, representing a significant safety benefit over conventional high‑dose growth factor delivery.

A disadvantage of PLA is that its hydrolytic degradation produces lactic acid, which may induce local inflammation and impair cell function and tissue repair [53]. BMP-2 also raises concerns regarding potential local immune reactions. Histological immunofluorescence staining of iNOS and CD163 was performed to preliminarily assess inflammatory responses and macrophage polarization states at the repaired site. The results showed only a minimal number of iNOS-positive cells at the interface in the sham and experimental groups (Figure 8C and G). The findings indicated limited macrophage aggregation, minimal M1 polarization and a low level of inflammatory response at the TBI and around the scaffolds. Previous studies have demonstrated that GM hydrogel exerts a mild immunomodulatory action that preferentially drives M2 macrophage polarization [54]. In the present study, the BMP-2/bFGF@GM-PLA scaffolds exhibited appreciably larger CD163-positive areas compared with the PLA scaffolds (Figure 8D and H), indicating enhanced M2 macrophage polarization. Integrating GM with the PLA fibers endowed the scaffold with a more favorable immune microenvironment for tissue repair and regeneration.

This study has certain limitations. First, the current study prioritizes phenotypic validation of LIPUS-stimulated scaffolds, demonstrating their potential to enhance rotator cuff healing. However, the molecular mechanism by which LIPUS and BMP‑2 coordinate to achieve a synergistic effect on proliferation and osteogenic differentiation remains incompletely defined. Future comprehensive investigations will optimize the combinatorial strategy for clinical applications and will be the primary focus of our subsequent research efforts. Also, the follow-up of 6 weeks is not representative of the long-term outcomes of rotator cuff healing. Extended-duration *in vivo* studies should be conducted to evaluate the long-term therapeutic efficacy, scaffold degradation, delayed heterotopic ossification risk and the persistence of hydrogel mediated M2 polarization. Furthermore, while the LIPUS parameters used in this study were effective, further optimization including safety and efficacy assessments is required for future clinical translation.

## Conclusion

This study developed BMP‑2/bFGF@GM‑PLA scaffolds by incorporating growth factor‑loaded hydrogel into PLA mats for rotator cuff repair. Under LIPUS stimulation, the scaffolds effectively promoted the recruitment, osteogenic and tenogenic differentiation of BMSCs. The external LIPUS stimulus played a pivotal role in integrating the mechanical and chemical cues of the composite scaffold, achieving a synergistic effect that enhanced TBI healing. Compared with single‑factor approaches, this innovative strategy highlights the significant potential of combining LIPUS with acoustic‑responsive biomaterial scaffolds for TBI regeneration and represents a promising approach for future clinical application.

## Supplementary Material

rbag112_Supplementary_Data

## Data Availability

The data that support the findings of this study are available on request from the corresponding author. The data are not publicly available due to privacy or ethical restrictions.
