# Supplementary material for: Low-intensity pulsed ultrasound stimulated hydrogel-polylactic acid composite scaffolds: a dual-cue approach for enhanced rotator cuff healing
Source: Regen Biomater. 2026 Jun 9;13:rbag112. doi: 10.1093/rb/rbag112 (PMC13344843; doi:10.1093/rb/rbag112)
Supplement: rbag112_Supplementary_Data [file rbag112_supplementary_data.zip › Supporting Information.docx]

**Supporting Information**

**Appendix Table 1.** The primers sequences of related genes used in RT-qPCR

| **Gene** | **Primer sequence (5’-3’)** |  |
| --- | --- | --- |
| RUNX2 | F: ATGCTTCATTCGCCTCACAAA  R: GCACTCACTGACTCGGTTGG | |
| OPN | F: AGCAAGAAACTCTTCCAAGCAA  R: GTGAGATTCGTCAGATTCATCCG | |
| SCX | F: TTCCCCGGTCCTAAAGGAAATG  R: GTGTCTCCTTTGTCACCACCA | |
| TNMD | F: GGGTGGTCCCGCAAGTGAAGGTG  R: CCTCGACGACAGTAAATACAACAGT | |
| GAPDH | F: AGAAGGCTGGGGCTCATTTG  R: GCAGGAGGCATTGCTGATGAT | |

**Appendix Table 2.** Histological Scoring System

| **Characteristic** | **Score** | | | |
| --- | --- | --- | --- | --- |
|  | **1** | **2** | **3** | **4** |
| Cellularity, % ^a^ | > 400 | 300 - 400 | 200 - 300 | < 200 |
| Vascularity ^b^ | > 15 | 10 - 15 | 6 - 10 | < 6 |
| Fiber orientation, % ^c^ | < 25 | 25 - 50 | 50 - 75 | > 75 |
| a. Number of cells per region of interest from each section, percentages represent relative values compared with the values from the normal tendon-bone sections, which were set at 100%.  b. Number of blood vessels per low power field (100-fold magnification) from each section.  c. Gray scale per region of interest from each section as measured by use of Image J, percentages represent relative values compared with the values from normal tendon-to-bone sections which were set at 100%. | | | | |

**
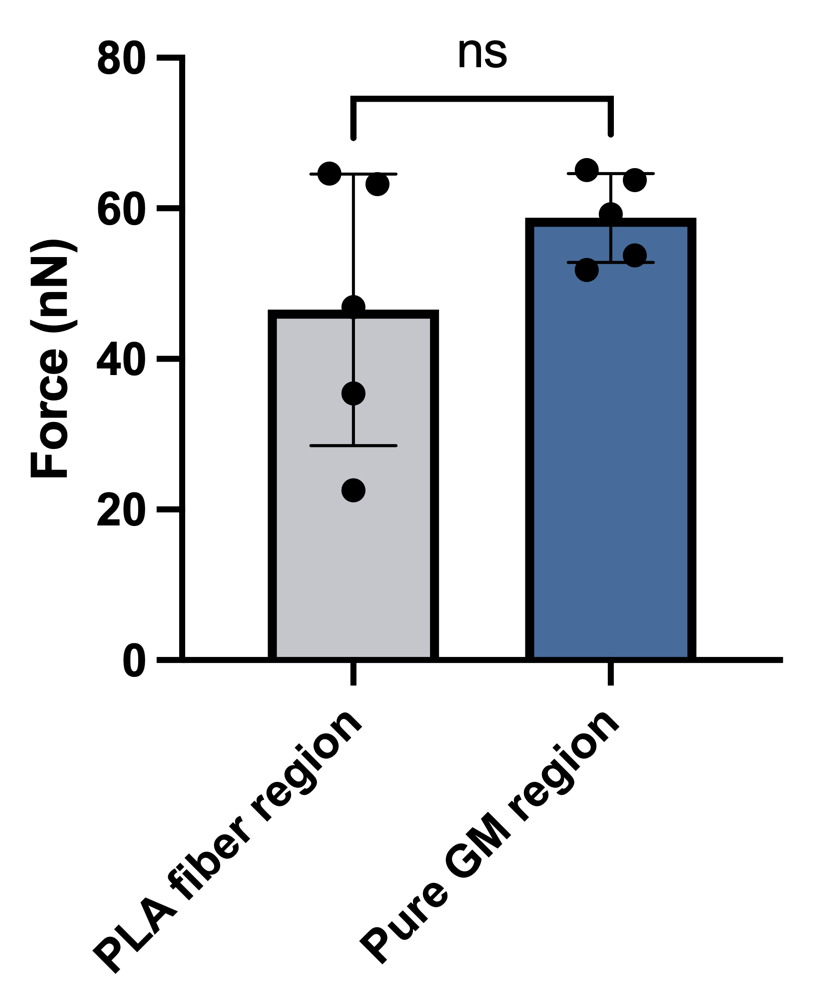
**

***Supplementary Figure 1.****Quantitative analysis of AFM pull-off force of the composite scaffolds after LIPUS stimulation (n = 5).*

**
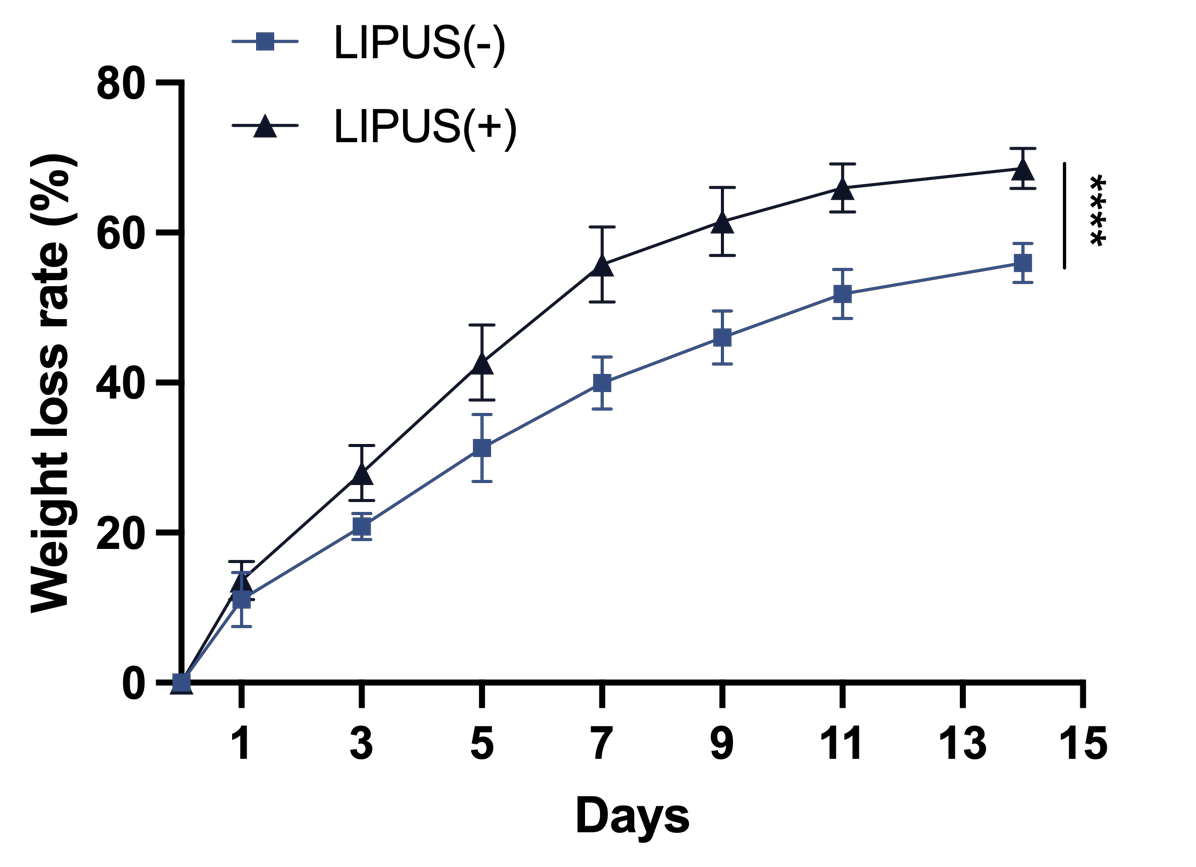
**

***Supplementary Figure 2.****Weight loss curves of the composite scaffolds at concentration of 6% with LIPUS stimulation (n = 3).*


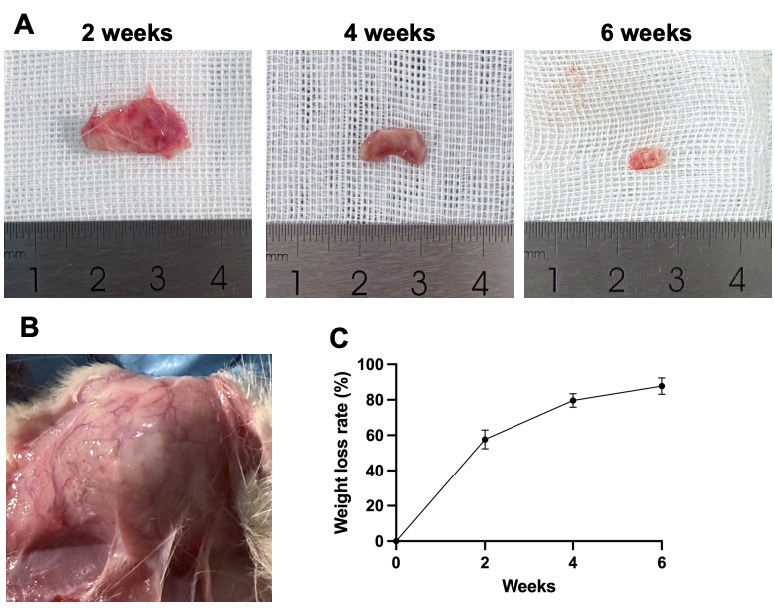


***Supplementary Figure 3.*** *Subcutaneous implantation of BMP-2/bFGF@GM-PLA scaffolds to evaluate the biocompatibility in vivo. (A) Gross observation of the retrieved scaffolds at different time intervals after implantation. (B) Representative images of scaffolds in the subcutaneous tissue. (C)Weight loss curves of the composite scaffolds in vivo.*

**
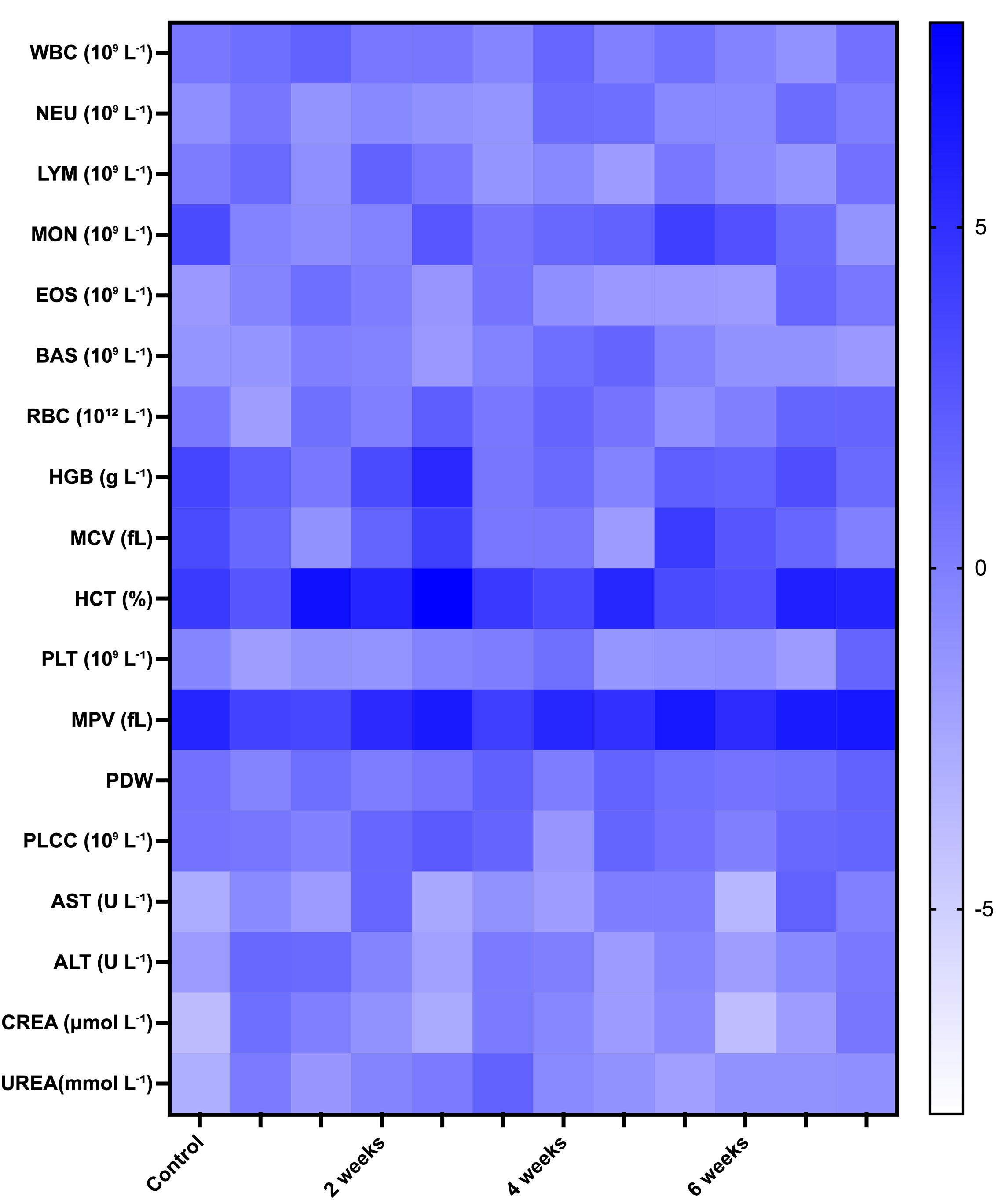
**

***Supplementary Figure 4.*** *Routine blood biochemistry analysis in rats sacrificed at different time intervals after implantation of BMP-2/bFGF@GM-PLA scaffolds (Data are presented as Z-score normalized values).*


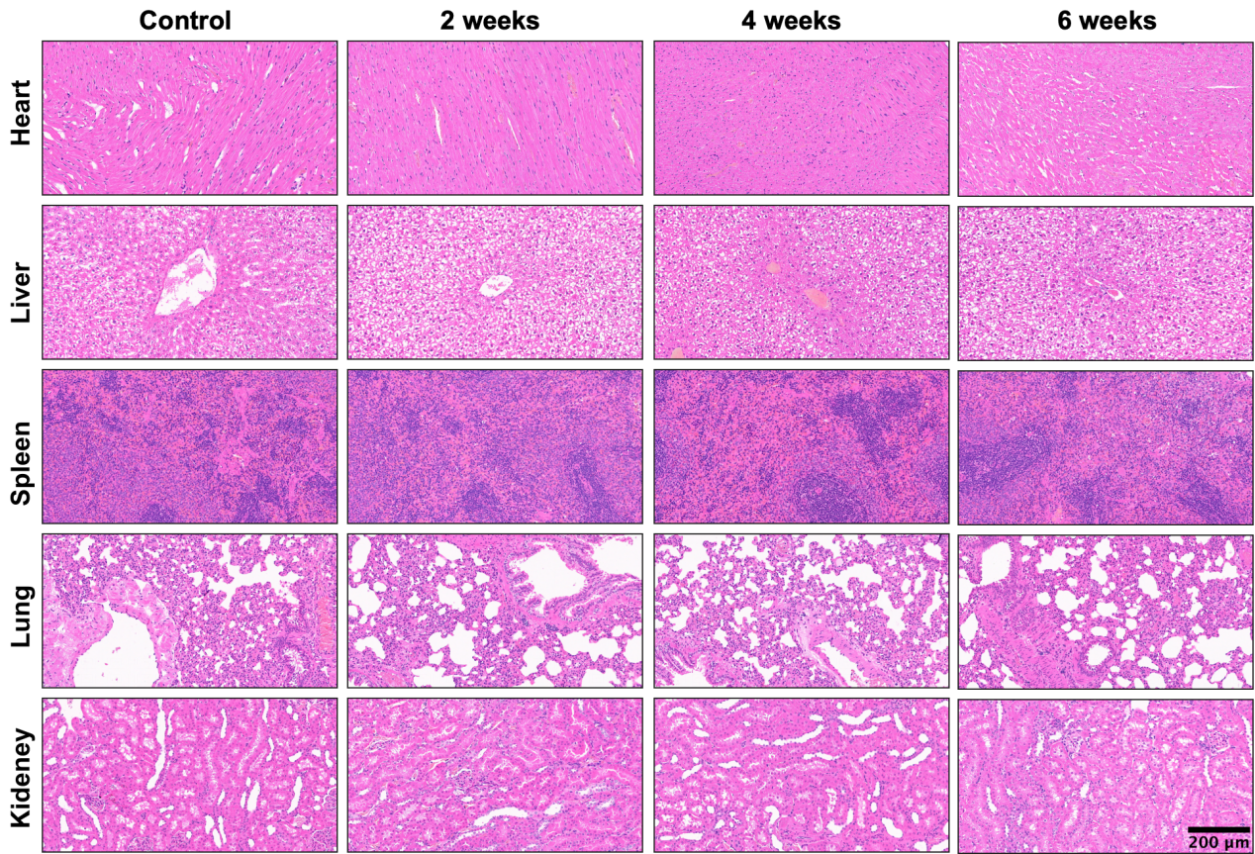


***Supplementary Figure 5.*** *HE staining of the major organs in rats sacrificed at different time intervals after implantation of BMP-2/bFGF@GM-PLA scaffolds.*

**
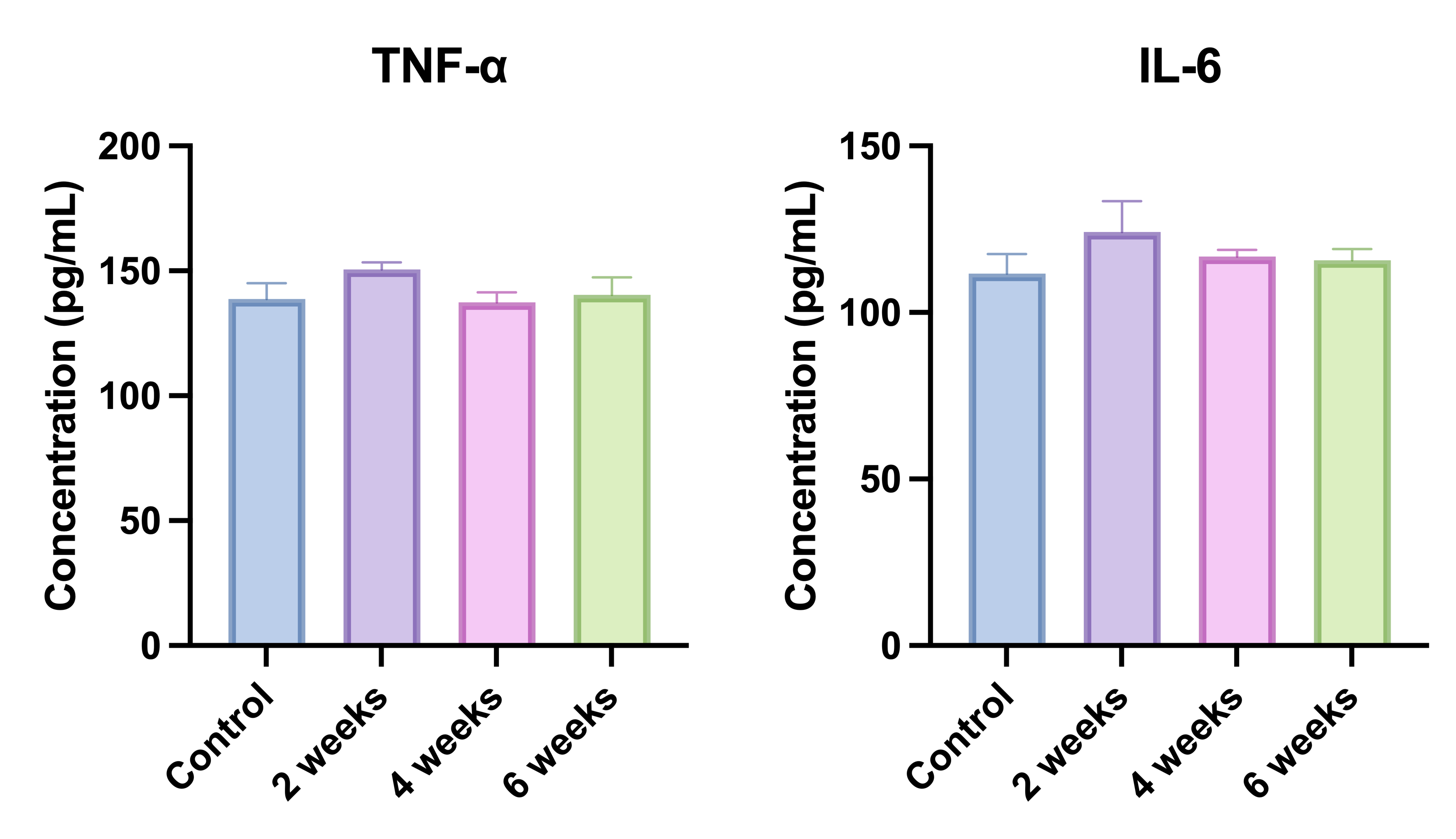
**

***Supplementary Figure 6.*** *Cytokine levels in surrounding tissue.*


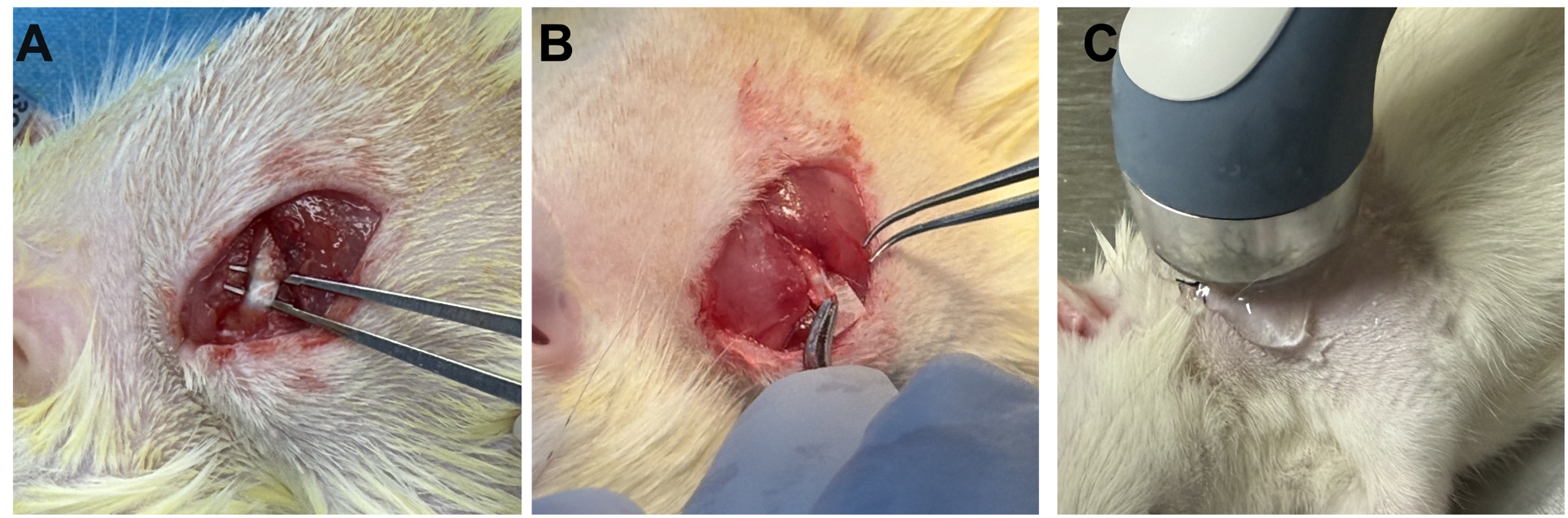


***Supplementary Figure 7.****Schematic diagram of implantation of scaffold in combine with post-operative LIPUS treatment for rotator cuff healing in vivo. (A) Exposure of the* *supraspinatus tendon* *insertion for transection during animal modeling. (B)* *Implantation of scaffold on the surface of the tendon insertion for transosseous suture with the* *tendon stump. (C) Daily postoperative LIPUS treatment at the repaired site.*
