# Supplementary figures and images for: Low-intensity pulsed ultrasound stimulated hydrogel-polylactic acid composite scaffolds: a dual-cue approach for enhanced rotator cuff healing
Source: Regen Biomater. 2026 Jun 9;13:rbag112. doi: 10.1093/rb/rbag112 (PMC13344843; doi:10.1093/rb/rbag112)

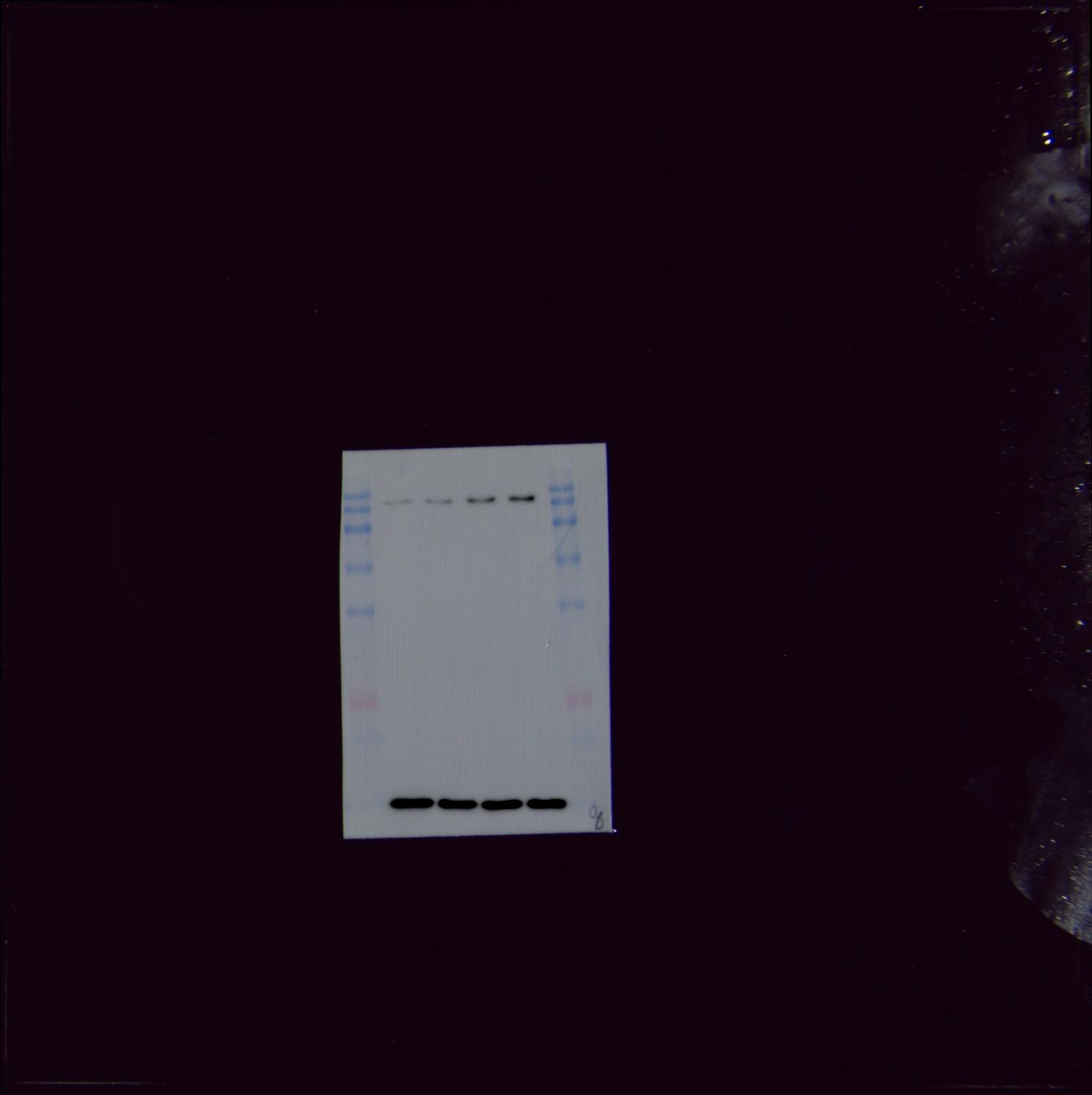

Supplement: rbag112_Supplementary_Data [file rbag112_supplementary_data.zip › Piezo1.jpg]

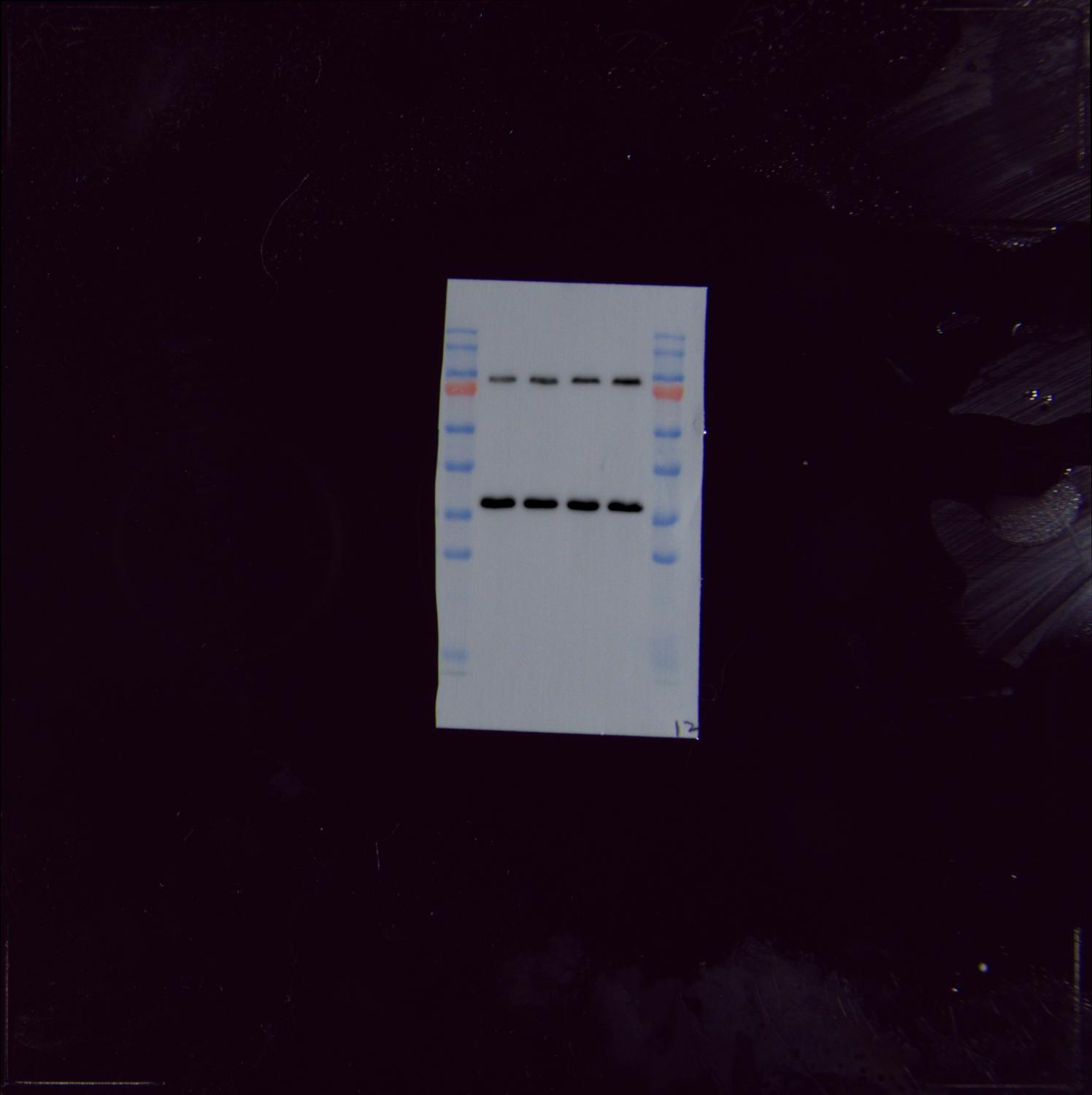

Supplement: rbag112_Supplementary_Data [file rbag112_supplementary_data.zip › β-Catenin.jpg]
